# Breeding Has Increased the Diversity of Cultivated Tomato in The Netherlands

**DOI:** 10.3389/fpls.2019.01606

**Published:** 2019-12-20

**Authors:** Henk J. Schouten, Yury Tikunov, Wouter Verkerke, Richard Finkers, Arnaud Bovy, Yuling Bai, Richard G.F. Visser

**Affiliations:** ^1^Plant Breeding, Wageningen University & Research, Wageningen, Netherlands; ^2^Business Unit Greenhouse Horticulture, Wageningen University & Research, Bleiswijk, Netherlands

**Keywords:** tomato varieties, diversity, introgressions, metabolomics, breeding

## Abstract

It is generally believed that domestication and breeding of plants has led to genetic erosion, including loss of nutritional value and resistances to diseases, especially in tomato. We studied the diversity dynamics of greenhouse tomato varieties in NW Europe, especially The Netherlands, over the last seven decades. According to the used SNP array, the genetic diversity was indeed very low during the 1960s, but is now eight times higher when compared to that dip. The pressure since the 1970s to apply less pesticides led to the introgression of many disease resistances from wild relatives, representing the first boost of genetic diversity. In Europe a second boost ensued, largely driven by German popular media who named poor tasting tomatoes *Wasserbomben* (water bombs). The subsequent collapse of Dutch tomato exports to Germany fueled breeding for fruit flavor, further increasing diversity since the 1990s. The increased diversity in composition of aroma volatiles observed starting from 1990s may reflect the efforts of breeders to improve fruit quality. Specific groups of aroma compounds showed different quantitative trend over the decades studied. Our study provides compelling evidence that breeding has increased the diversity of tomato varieties considerably since the 1970s.

## Introduction

A recent paper in *Nature Biotechnology* on *de novo* domestication of tomato voiced the general belief that “breeding of crops over millennia for yield and productivity has led to reduced genetic diversity. As a result, beneficial traits of wild species, such as disease resistance and stress tolerance, have been lost (…). Despite the increases in yield conferred by domestication, the breeding focus on yield has been accompanied by a loss of genetic diversity and reduced nutritional value and taste” (Zsögön, 2018).

Reduction of diversity among crop varieties poses risks for cultivation, especially when most varieties carry the same genetic basis for resistance to diseases and pests. If a disease resistance is overcome in one variety, other varieties become susceptible too. This leads to agricultural vulnerability which can affect the entire chain, especially if there are no alternatives for disease control, such as appropriate, authorized pesticides. History has provided several examples, such as the Panama disease (Fusarium oxysporum f. sp. cubense) epidemic in banana (Ploetz, 2000; Garcia, 2013), or the southern corn leaf blight (*Helminthosporium maydis*) outbreak in maize (Horsfall, 1972; Kemble et al., 1982). Because the number of authorized pesticides has decreased and continues to decrease, crop protection has to rely more and more on resistances that should have not a narrow genetic basis.

The loss of genetic variation in crops due to the modernization of agriculture has been denoted as genetic erosion (Tanksley and McCouch, 1997). During domestication preferred genotypes were selected, leading to loss of alleles and a decrease in genetic diversity of landraces compared to wild accessions (Bai and Lindhout, 2007; Blanca, 2015; Lin et al., 2014). Two principal occurrences affecting crop diversity have been identified: 1) the replacement of landraces by commercial varieties; and 2) more recent additional changes in the diversity of commercial varieties caused by plant breeding (van de Wouw et al., 2010a). Breeding can reduce genetic diversity by continued selection in the breeding germplasm, or may broaden genetic diversity through the introgression of alleles from wild relatives. The question remains whether the increase in diversity because of introgression has compensated the reduction of genetic diversity due to inbreeding and selection.

We have studied this for tomato, as particularly in this crop there have been indications of serious genetic erosion (Lin et al., 2014). Furthermore, Tieman and Klee (Tieman, 2017; Klee and Tieman, 2018) mentioned that “modern commercial varieties contain significantly lower amounts of many (…) important flavor chemicals than older varieties” as a result of intensive selection for production traits, such as yield and disease resistance, at the expense of flavor. We studied the evolution of diversity of commercial tomato varieties in NW Europe since the 1950s. To do this, we looked at both genetic variation at the DNA level, and phenotypic variation, including disease resistances, fruit size, and flavor components.

## Materials and Methods

### Tomato Varieties and Growing Conditions

Ninety tomato varieties introduced in the Netherlands for commercial glasshouse fresh fruit production in a time period from 1950 till 2016 were selected by random picking without any prior knowledge about any of genetic or phenotypic parameters analyzed, to have about 12 varieties per each decade (**Table S1**). Although for recent decades far more varieties were available compared to the 1950s, we decided to have a balanced sampling with similar sampling sizes for the different decades, thus preventing changes in diversity due to differences in number of sampled varieties per decade. The varieties were grown in a glasshouse at standard commercial growing conditions in the summer of 2017. Three plants per variety were grown using a randomized block experimental design.

### Genetic Diversity Analysis

Young leaf material was collected from the 90 tomato varieties, freeze dried, and sent to Trait Genetics, Germany, for genotyping by means of a Illumina^®^ SolCAP SNP-array (Sim, 2012a). This yielded 7720 SNP-marker scores per variety. The SNP-scores were visualized in Excel, using a blanc cell in case the score resembled the reference genome. In addition to the SNP array genomic DNA from two recent varieties from this list, i.e. Merlice and Bambelo, was re-sequenced using Illumina HiSeq 150 paired ends sequencing, for detailed analysis of introgressions.

### Genetic Diversity Index Calculation

Within each decade we calculated for each SNP the nucleotide frequencies among the varieties that were commercially introduced in that decade, and used these frequencies for calculating the genetic diversity index (*H*) of Nei (1973), according the equation:

$$H=1-\sum_{i}p_{i}^{2}$$

where: *p* = frequency of nucleotide *i*.

This is also named “expected heterozygosity.” The *H*-values per decade were averaged among all markers, giving a measure for genetic diversity for each decade. The frequency *p* was also calculated at the diploid level, looking at frequencies of allelic combinations in commercial varieties.

### Definition of Basal Genome and Introgressions

For finding introgression that were deliberately introduced by breeders, we needed an introgression free reference genome. The generally used reference genome of tomato refers to cv. Heinz. However, this variety may harbor introgressions. Therefore, we defined a “basal genome,” which is the consensus genome of the sampled varieties from the 1950s and 1960s. For each SolCap array marker, we selected the most common nucleotide in these two decennia. Marker scores that deviated from this basal genome were highlighted in Excel, using conditional formatting. This revealed introgression haploblocks.

### Analysis of the Basic Fruit Flavor Parameters

For the analysis of the basic flavor parameters: soluble solids content (Brix), titratable acidity, firmness, and juiciness from 20 to 30 fruit per variety were harvested per a variety at a mature ripe stage. Fruit ripening was judged using intensity of pigmentation and firmness. Also average fruit weight was recorded for every variety analyzed.

For soluble solids content and titratable acidity measurements, fruit quarters (for fruits >30 g per fruit) and whole fruits (<30 g per fruit) were homogenized for 15 s in a Vita-prep 3 blender. The soluble solids content was measured directly from the homogenized fruit sample as °Brix by means of a Refracto 30PX digital refractometer (Mettler Toledo). Titratable acidity (mmol H3O+/100 gram fresh weight) was determined by means of potentiometric endpoint titration with 0.1 mol/l NaOH till pH 8.2 by means of a T50 titrator (Mettler Toledo).

For texture measurements, from each fruit a 10-mm-diameter disk was excised from the fruit pericarp at the locular region by means of a cork borer. The disks were patted dry by rolling on filter paper and were weighed on an analytical balance (Mettler Toledo XA 204 DeltaRange). Five disks were enclosed between sheets of screening cloth (Agratex, Ludvig Svensson), placed between two sheets of pre-weighed filter paper, having the skin down (Whatman 1003-917) and compressed by means of an Instron 3343 Universal Testing Machine at a speed of 60 mm/min with a flat plate plunger to 900 N. After this compressing, the filter paper was re-weighed. The juiciness (% Juice) was calculated as the weight increase of the filter papers divided by the fresh weight of the disks. Pericarp firmness was defined as the force [Newton (N)] at break of the force/deformation curve of the five simultaneously compressed disks. The data of the basic flavor parameters are present in **Supplemental Data (Data Sheet S7)**.

### Analysis of Volatile Compounds

Nine mature ripe fruits per variety were pooled to have a representative sample. Fruits were cut, immediately frozen in liquid nitrogen and were ground to fine powder under liquid nitrogen using A11 analytical mill (IKA). Volatile organic compounds (VOCs) were analyzed, identified, and quantified using a Solid Phase Micro Extraction–Gas Chromatography–Mass Spectrometry (SPME-GC-MS) method previously described (Tikunov, 2005; Tikunov, 2013). Frozen fruit powder (1 g fresh weight) was weighed into a 5-ml screw-cap vial, closed, and incubated for 10 min at 30°C. An aqueous EDTA-sodium hydroxide (NaOH) solution was prepared by adjusting 100 mM EDTA to pH of 7.5 with NaOH. Then, 1 ml of the EDTA-NaOH solution was added to the sample to give a final EDTA concentration of 50 mM. Solid CaCl_2_ was immediately added to give a final concentration of 5 M. The closed vials were sonicated for 5 min. A 1-ml aliquot of the pulp was transferred into a 10-ml crimp cap vial (Waters), capped, and used for SPME GC-MS analysis. Volatiles were automatically extracted from the vial headspace and injected into the GC-MS *via* a Combi PAL autosampler (CTC Analytics). Headspace volatiles were extracted by exposing a 65-µm polydimethylsiloxane-divenylbenzene SPME fiber (Supelco) to the vial headspace for 20 min under continuous agitation and heating at 50°C. The fiber was desorbed in the GC-MS injection port and compounds were separated on an HP-5 (50 m × 0.32 mm × 1.05 µm) column with helium as carrier gas (37 kPa). Mass spectra in the 35 to 400 m/z range were recorded by an MD800 electron impact MS (Fisons Instruments) at a scanning speed of 2.8 scans/s and an ionization energy of 70 eV. The chromatography and spectral data were evaluated using Xcalibur software (Thermo Scientific). MSClust software (Tikunov et al., 2011) was used to extract volatile compound mass spectral information from the chromatograms. Forty-six compounds were identified by comparison with authentic chemical standards (**Data Sheet S5**); the other were tentatively identified using MSSearch software (Thermo) and the NIST mass spectral library (www.nist.gov). 

### Analysis of Flavor Diversity

To estimate time trends of basic flavor components and individual volatile compounds, the Mann-Kendall trend test (Gilbert, 1987) implemented in Past3 software (https://folk.uio.no/ohammer/past/) was used. To discover trends in the diversity of volatile compound composition, a pairwise Euclidean distances matrix was calculated based on the quantitative profiles of 69 annotated volatiles (**Data Sheet S5**). Then a mean distance was calculated for each variety by averaging its distances to all other varieties registered within ±5 years. These mean distances of all 90 varieties were subjected to the Mann-Kendall trend test.

## Results and Discussion

### Genetic Diversity of Tomato Varieties During the Previous Seven Decades

**Figure 1:**
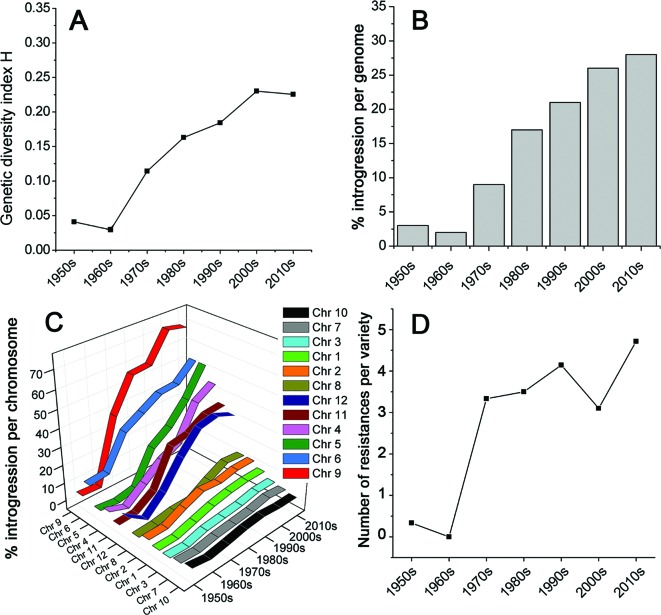
Genetic diversity of tomato varieties, commercially introduced from 1950 till 2016. **(A)** The diversity within a decade is expressed as Nei’s index, also referred to as expected heterozygosity. **(B)** The proportion of the genome and **(C)** individual chromosomes of commercial tomato varieties, consisting of introgressions compared to the prevailing genome of tomato varieties in the 1950s and 1960s. **(D)** Average number of diseases and pest to which the investigated varieties are resistant, according to the official NAKTuinbouw variety database [https://www.naktuinbouw.com/groente/variety-description/tomato-solanum-lycopersicum-l; (Banga and Aalbersberg, 1995)].

We collected leaf samples from 90 tomato varieties that were commercially released between 1950 and 2016 in NW Europe (**Table S1**). All varieties have been used in greenhouses for the production of tomatoes for the fresh market. We analyzed roughly equal numbers of varieties per decade from the 1950s till the 2010s. The varieties were genotyped using the SolCap SNP platform (Sim, 2012a). This yielded 7720 SNP-marker scores per variety (**Data Sheet S1**). SNP-markers containing missing values over the majority of varieties were removed, leaving 7,661 SNP-markers. We grouped the varieties per decade and calculated for each SNP the genetic diversity index (*H*) of Nei (Nei, 1973), also referred to as expected heterozygosity. These *H*-values per decade were averaged over all markers, providing a measure for the genetic diversity of each decade (**Figure 1**). This figure clearly shows that the genetic diversity among commercial tomato varieties was low during the 1950s, and even lower during the 1960s. However, from the 1970s onwards, the diversity increased up to eight-fold compared to the 1960s, according to the studied SNPs, and using the diversity index *H*. Apparently, the increase in diversity caused by introgressions far exceeded the decrease in diversity caused by selection.

**Figure 2:**
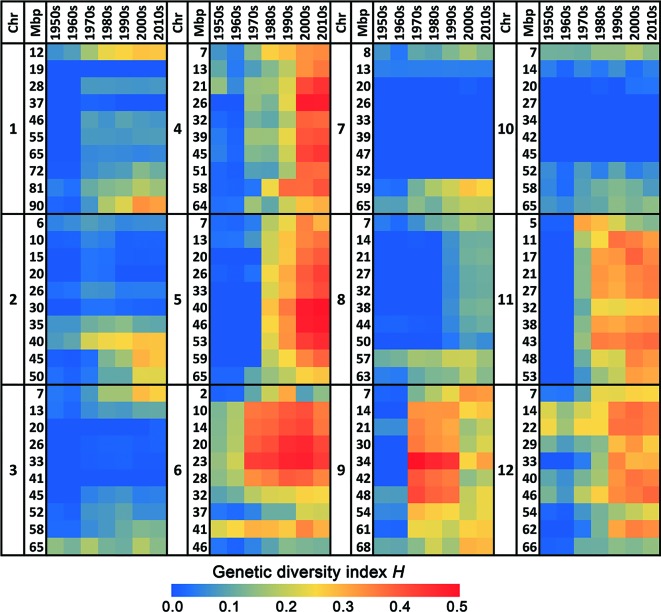
Heat map of the genetic diversity in the 12 chromosomes of commercial tomato varieties in course of time. The blue color indicates a very low genetic diversity in that decade for the respective chromosomal fragment, whereas a red color represents a very high genetic diversity.

This increase in genetic diversity in recent varieties compared to the low diversity in those from the 1950s and 1960s is not restricted to a few loci only, but has occurred across the whole genome (**Figures 1** and **2**). Chromosomes 4, 5, 6, 9, 11, and 12 show a particularly pronounced increase in diversity in modern varieties. Apparently, the increased genetic diversity has not been limited to a small number of genes or a few chromosome arms, but has encompassed the majority of all tomato genes. However, some regions have hardly changed since the 1950s, e.g., the upper half of Chr. 2, harboring repeats of 45S ribosomal DNA (The Tomato Genome Consortium, 2012), and the central parts of Chrs. 3, 7, and 10 (**Figure 2**), being the centromeric regions of these chromosomes (Víquez-Zamora, 2014).

As **Figure 1** shows, genetic diversity was very low in the 1950s and 1960s. Based on this observation, we defined a “base tomato genome,” representing for each SNP-marker the most prevalent nucleotide during these two decades. The nucleotides deviating from this “base genome” were regarded as introgressions. **Figure 1** illustrates the gradual increase in the proportion of introgressed DNA. Currently, more than a quarter of the genome (28%) is composed of such introgressions.

Before 1970, nearly all tomato varieties were homozygous, but from 1980 onwards, nearly all new commercial varieties are hybrids (**Figure S1**), giving an extra layer of genetic diversity, i.e., within the varieties. In **Figures 1** and **2**, we did not separate heterozygous from homozygous introgressions, giving heterozygous introgressions the same weight as homozygous ones. However, in **Data Sheet S3**, we show the separate introgressions in the individual varieties, distinguishing homozygous from heterozygous haploblocks. **Data Sheet S3** clearly show the increase in abundancy of haploblocks from the 1970s onwards. Several of these haploblocks are very large, reflecting linkage drag. The “basal genome” without deliberate introgressions is shown in **Data Sheet S3** too. This basal genome consists of the consensus marker scores of the varieties sampled in the 1950s and 1960s.

### The First Diversity Boost: Introgressions for Resistances

The chromosomes differed considerably in their introgression composition (**Figure 1C**). Two groups of chromosomes can be distinguished. In the first group of chromosomes (Chr. 1, 2, 3, 7, 8, 10), only 5 to 15% of the chromosomal DNA has been altered since the 1960s. In the second group (Chr. 4, 5, 6, 9, 11, 12), between 30 and 70% of the chromosome has been replaced by introgressions. There is one chromosome (Chr. 9) that consists of approximately 70% introgressed DNA, compared to the 1960s.

This huge change in the composition of Chr. 9 (**Figure 1C**) was caused by a large introgression fragment from *Solanum peruvianum*. This introgression carries the tomato mosaic virus (ToMV) resistance gene *Tm2* (derived from *S. peruvianum* PI 126926) or its allele *Tm2* *^2^* (derived from *S. peruvianum* PI 18650) (Lanfermeijer et al., 2003; Lin et al., 2014). We re-sequenced the recently (2013) introduced variety “Merlice,” being homozygous for *Tm2* *^2^* (**Data Sheet S2**). The exotic fragment encompasses 79% (53 Mb) of Chr 9 in this modern variety (**Figure S2**). Breeding companies started selling tomato varieties with this introgression in the 1970s, and the proportion of varieties carrying this introgression has increased ever since (58% in the 1970s to 93% in the 2010s; **Figure S3**). However, the introgression size has remained large, showing the co-introgression of the majority of Chr. 9 from the wild species [**Data Sheet S3**, (Víquez-Zamora, 2013; Lin et al., 2014)]. This linkage drag has likely been caused by recombination suppression during meiosis, possibly due to large structural rearrangements such as an inversion in this region (Bonierbale et al., 1988).

From the 1970s onwards, the genes *Cf-4* and *Cf-9* were introgressed at the top of chromosome 1, for providing resistance to leaf mold disease, caused by *Cladosporium fulvum* (**Data Sheet S6**). These genes descend from *S. pimpinellifolium* (**Data Sheet S6**). Two other resistance genes for controlling this pathogen, i.e., *Cf-2* and *Cf-5*, were introgressed at the top of chromosome 6. These genes were introgressed since the 1970s too.

Another resistance gene on chromosome 6, introgressed during the same period, is the *Mi-1* gene from *S. peruvianum*, conferring resistance to southern root-knot nematode (*Meloidogyne incognita*). This introgression has remained very large since its introgression, covering nearly 60% of the chromosome [**Data Sheet S6**; (Víquez-Zamora, 2013; Lin et al., 2014)]. Many more resistance genes have been introgressed since the 1970s, on nearly all chromosomes of tomato (**Data Sheet S6**).

Analysis of the phenotypic traits of the varieties since the 1960s, as described by descriptive variety lists (Banga and Aalbersberg, 1995), showed that also at the phenotypic level, the diversity among varieties was very small in the 1960s, but from the 1970s onwards, an increasing number of resistances to diseases and pests were introduced (**Figure 1D**, **Data Sheet S4**).

### The Second Diversity Boost: Fruit Quality Traits; “Discharging Water Bombs”

Poor flavor is the most frequent reason for consumer dissatisfaction of tomatoes (Fernqvist and Hunter, 2012). In the late 1980s and early 1990s, German popular news media coined the phrase *Wasserbomben* (German for “water bombs”) to describe poor, watery-tasting Dutch tomatoes (Hendriks, 2016). At that time, the vast majority of Dutch fresh tomatoes were being exported to Germany. Following the damage to their reputation, Dutch tomato exports to Germany collapsed shortly after (Hendriks, 2016). This became an important milestone in tomato breeding in NW Europe, marking the need for an adjustment of selection criteria towards balancing agronomical traits, such as high yield and resistances, with consumer quality traits, such as flavor, fruit size, shape, and color. Also, research on better and quicker measurement techniques of flavor started. The *Wasserbomben* crisis fueled the second boost of diversity, namely a diversity in fruit types and improved flavors.

### Fruit Size

**Figure 3:**
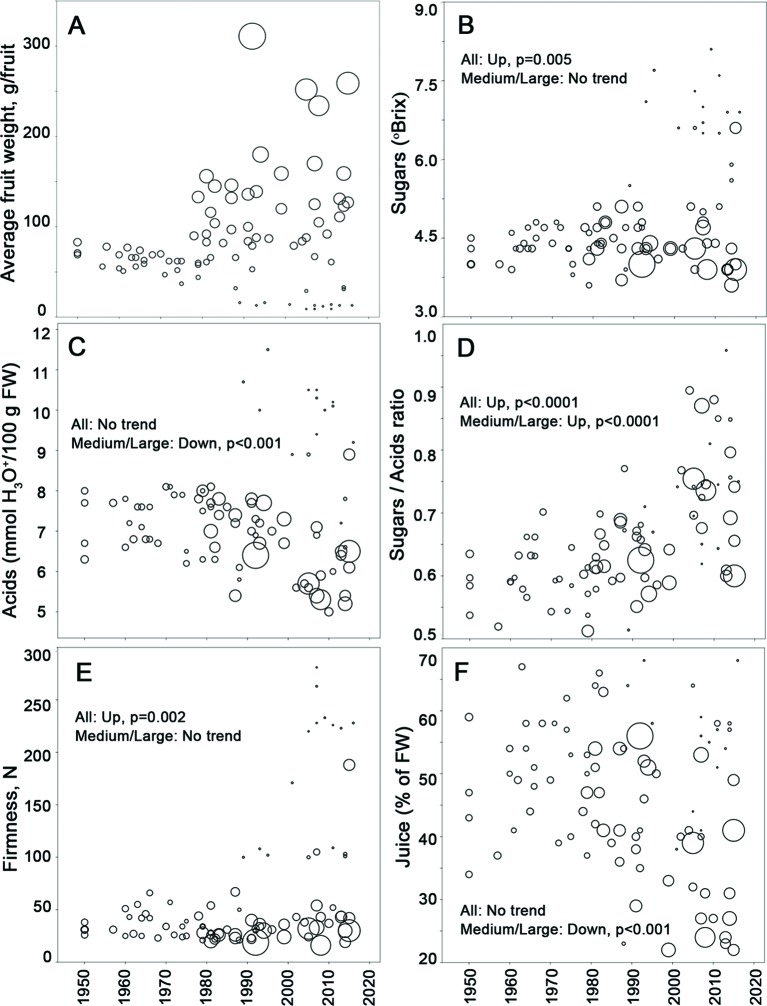
The development of average fruit weight and the basic fruit flavor parameters of tomato varieties, commercially introduced between 1950 and 2016. Each variety is represented by a circle, whose size is proportional to its average fruit weight. Significance of quantitative trends is indicated with p-values of Mann-Kendall trend test for all varieties analyzed (All) and for medium-sized and large fruited tomatoes with average weight >25 g per fruit (Medium/Large). **(A)** Average fruit weight, g/fruit, **(B)** Sugar content in ripe fruit measured as refraction index, ºBrix, **(C)** Titratable acids in ripe fruit, mmol H_3_O^+^/100 g FW, **(D)** Sugar/Acid ratio in ripe fruit, **(E)** Firmness of ripe fruit, N, **(F)** Amount of liquid (juice) released by ripe fruit, % of FW.

The most obvious phenotypic diversification that occurred from the 1980s onwards has been fruit size (**Figure 3A**). While varieties that entered the market before 1980 showed little variation in average weight, ranging predominantly between 50 and 100 g per fruit, the introduction of new fruit types, such as cherry and cocktail tomatoes (<25 g per fruit) as well as large fruited varieties (100–300 g) led to an enormous diversification of fruit sizes from the 1980s onwards (**Figures 3A** and **S4C**). Víquez-Zamora et al. (2013) showed that the cherry-like fruit sizes were obtained by introgressions of large parts of Chrs. 4, 5, and 12 from *S. pimpinellifolium*. This is consistent with the increased diversity of these chromosomes since the 1990s, apparent from the heat map in **Figure 2**.

### Flavor

Tomato fruit flavor is determined by a combination of five essential chemical and textural components: 1) the concentration of sugars in ripe tomato, mainly fructose and glucose, which can be very well approximated by soluble solids content, measured as a refraction index (°Brix); 2) acidity, which is determined by the concentration of organic acids, mainly citrate; 3) firmness of the fruit pericarp; 4) fruit juiciness; and 5) aroma, caused by a complex combination of VOCs. The ratio of the first two components—sugar content and acidity—is one of the main parameters for the perception of sweetness (Tandon et al., 2003; Zanor, 2009). The sugar/acid ratio showed an overall significant increase in the last three decades (**Figure 3D**). On the one hand, cherry tomatoes with a higher sugar content were introduced in that period (**Figure 3B** and **Table S2**). Concurrently, there was a reduction in the acidity of medium-sized and large fruits (**Figure 3C**). Fruit pericarp firmness has increased mainly due to the introduction of cherry varieties (**Figure 3E**). Variation in juiciness appears to have been considerable in all decades since 1950, although interestingly, the proportion of varieties whose fruit tissue released less liquid has increased during the last three decades (**Figure 3F**). These may represent an increasing interest in “non-leaky” varieties for use in salads and on bread.

**Figure 4:**
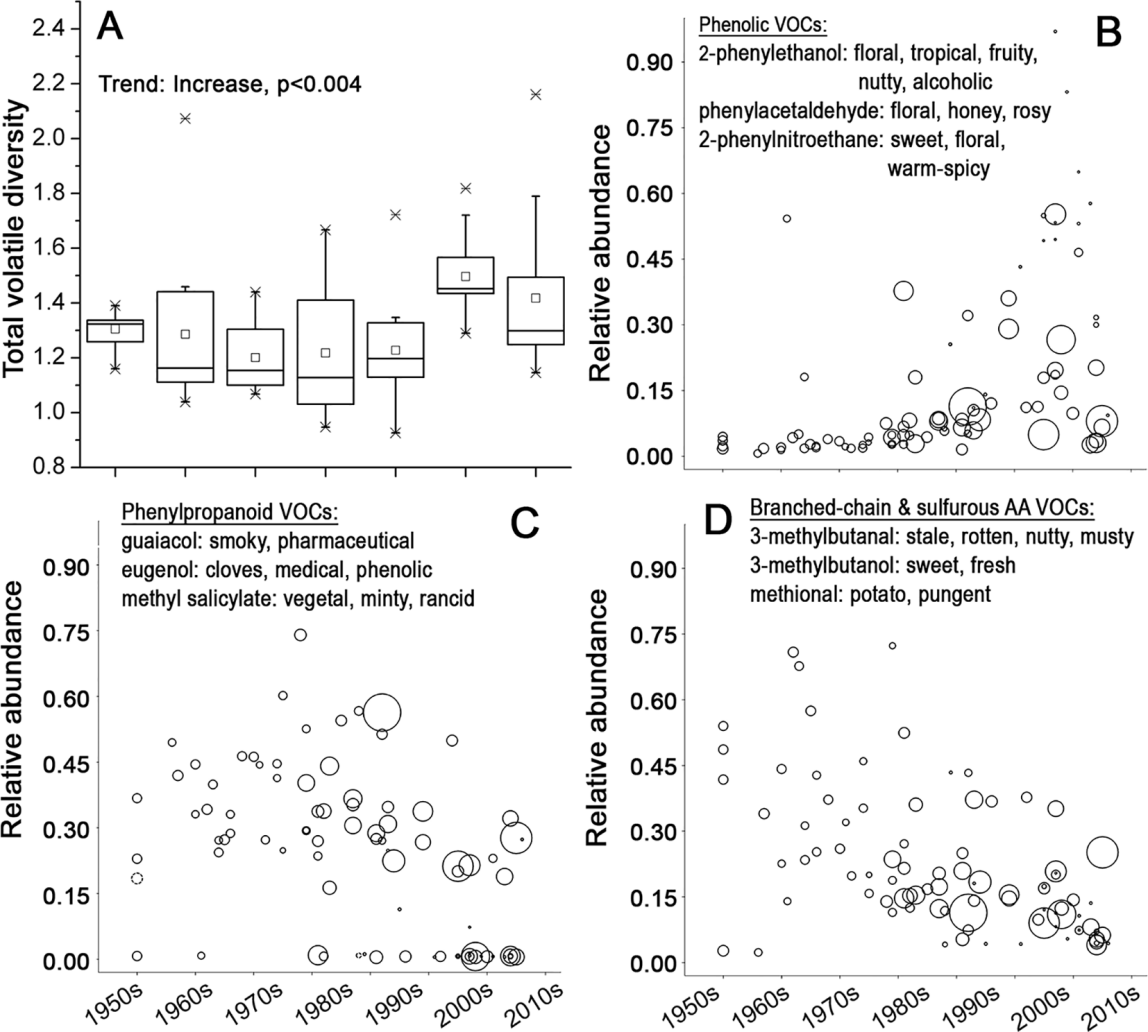
The composition of volatiles in tomato fruits in course of time. **(A)** Boxplot of the quantitative diversity of volatiles of fruits of tomato varieties in the respective decades. The diversity is expressed as Euclidian distances between quantities of 60 identified volatile compounds (VOCs). **(B**–**D)** Relative average abundance of three VOC groups with their individual aroma characters. Each variety is represented by a circle, whose size is proportional to the average fruit weight. **(B)** Phenolic VOCs, **(C)** phenypropanoid VOCs, and **(D)** VOCs derived from branched-chain and sulfurous amino acids in fruits of 90 tomato varieties.

Most of the aroma active VOCs in tomato fruit can be classified into five distinct groups according to their biosynthetic origins (Rambla et al., 2014): 1) VOCs derived from fatty acids; 2) phenolic VOCs; 3) phenylpropanoid VOCs; 4) VOCs derived from sulfur-containing and branched chain amino acids; and 5) carotenoid breakdown products (Buttery et al., 1987; Buttery et al., 1988; Krumbein and Auerswald, 1998; Baldwin et al., 2000; Tandon et al., 2000; Tandon et al., 2001; Selli et al., 2014; Du et al., 2015; Tieman, 2017). Overall, the VOC composition of tomato fruits has diversified significantly, particularly over the last two decades (**Figure 4A**). However, different chemicals with distinct aroma characteristics showed different temporal trends (**Table S3**). The second group of phenolic VOCs associated with floral and sweet aroma (Tandon et al., 2000; Baldwin et al., 2008; Mayer, 2008; Selli et al., 2014; Du et al., 2015) increased dramatically in the fruits of more modern varieties when compared to earlier varieties (**Figures 4B** and **S5**). The third group of phenylpropanoid VOCs, which express a “smoky” (Tikunov, 2013) or “medical” aroma (Causse, 2002), and the fourth group of VOCs produced from branched-chain and sulfurous amino acids, associated with earthy/musty/pungent/medicinal types of aroma (Baldwin et al., 2004; Baldwin et al., 2008), showed a very significant reduction in fruits of the more recent varieties (**Figures 4C, D**, **Figure S5** and **Table S3**).

Tomato fruit flavor is a product of a complex interaction between all the mentioned factors (Baldwin et al., 2008). Our data indicate that breeding has led to a clear increase in variation of different flavor components from the beginning of 1990s. This can be regarded as the second boost of diversity, since it began two decades later than the general increase in genetic diversity. More specifically, there has been an increasing proportion of varieties with a higher potential to express sweet/fruity types of flavor along with reduced expression of potential off-flavors.

Some recent studies, such as a work of Tieman and Klee (Tieman, 2017; Klee and Tieman, 2018), comparing modern commercial varieties to old, non-commercial heirloom tomatoes, conclude that the modern varieties had a lower flavor quality compared to the old varieties due to breeding. This seeming discrepancy with our findings might be due to the difference in the definition of “old,” which in Tieman et al. study refers to tomato varieties that were not subjected to breeding for traits relevant for industrial tomato production, and some of them may originate from long before 1950s. In our collection, the oldest varieties of 1950–1960s are all indeterminate greenhouse varieties that already went through the breeding for the production-related traits, which make their genetic diversity much narrower compared to the heirlooms. Secondly, our modern varieties have been bred for greenhouses in NW Europe, whereas Tieman and Klee studied open field varieties for N. America.

Considering the existence of other sources of biodiversity, e.g., landraces or heirloom varieties predating the 1950s and wild relatives, there may still be room for improvement of flavor of modern commercial tomatoes by further enrichment with alleles that can further improve fruit flavor (Tieman, 2017). However, our data indicate that modern breeding for quality for the consumer is on the right track. Pre-harvest, harvesting, and post-harvest practices are other important factors affecting variation in flavor quality. Therefore, breeding for and introduction of superior genotypes must be complemented by appropriate growing and post-harvest practices to ensure the maximum translation of genetics into flavor of desirable quality, and to deliver it to consumers at its best.

### Diversity Evolution in a Wider Temporal Context

In our analysis, we looked at varieties from the 1950s till the present time, reflecting the period of modern commercial tomato breeding. In order to put the evolution of diversity in a wider temporal context, we calculated the diversity index among 385 ancestors (*S. lycopersicum* var. *lycopersicum*, *S. lycopersicum* var. *cerasiforme*, and *S. pimpinellifolium*), and 129 vintage accessions (including landraces and heirlooms). We used the SolCap array data and the classification of genotypes from Blanca et al. (2015), selecting the same SNP-markers as were used for **Figure 1**.

**Figure 5:**
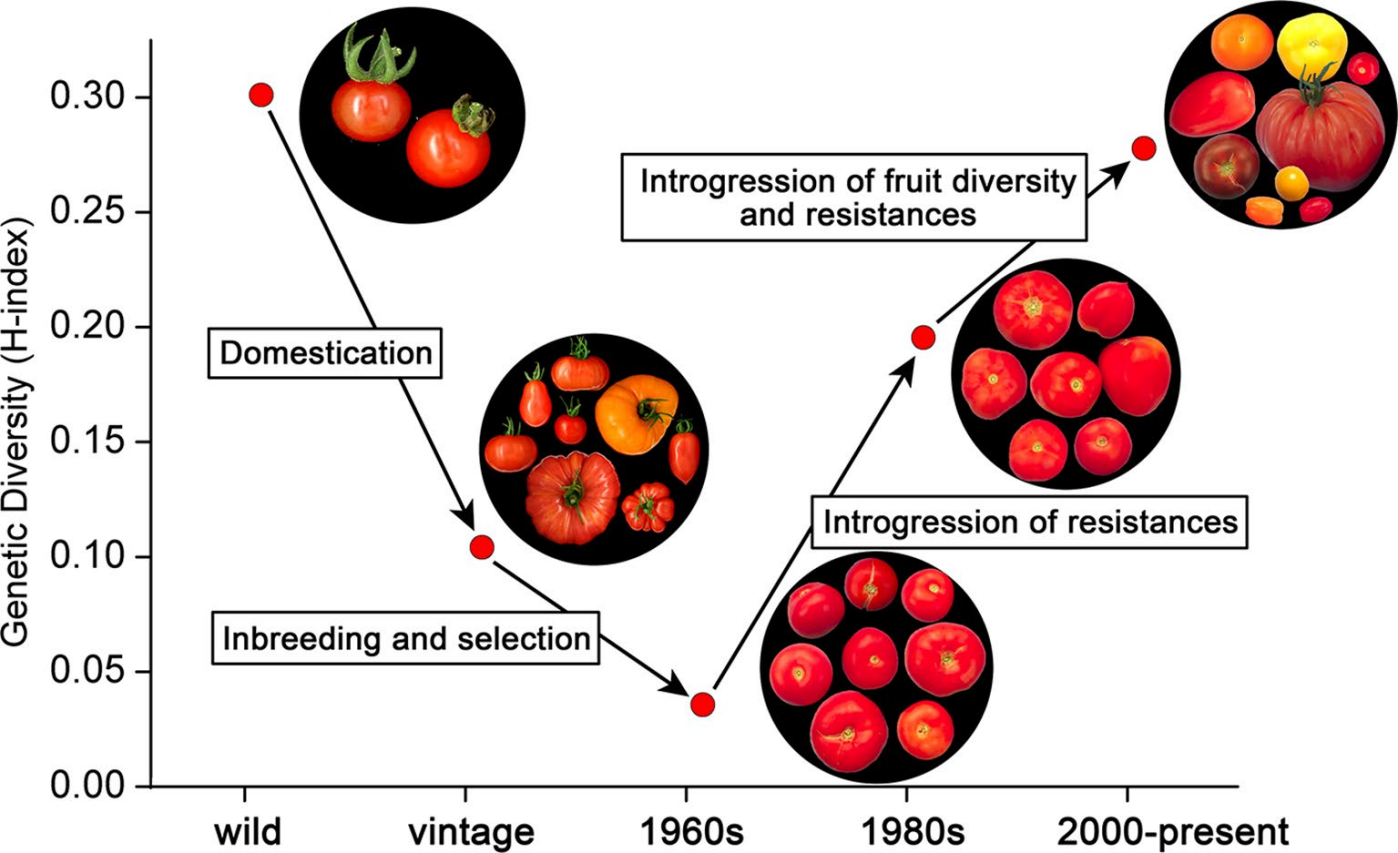
The evolution of genetic diversity in tomato. The upper left group represents the ancestors (*S. lycopersicum* var. *lycopersicum*, *S. lycopersicum* var. *cerasiforme*, and *S. pimpinellifolium*), which gave rise to the vintage types including landraces through a process of domestication [data from (Blanca, 2015)]. Inbreeding and selection among these vintage tomatoes led to commercial varieties in the 1960s with a very low genetic and phenotypic diversity. From the 1970s onwards, resistances to diseases and pests were introgressed from distant species, including *S. peruvianum*, *S. pennellii*, *S. chilense*, and *S. habrochaites*, increasing genetic diversity among commercial tomato varieties considerably. After the 1980s, fruit size, color, and flavor started to vary substantially, further increasing the genetic diversity of modern varieties.

**Figure 5** shows the relatively high diversity among ancestors belonging to *S. lycopersicum* and *S. pimpinellifolium*. Domestication of these species and genetic bottlenecks due to transport (Blanca, 2012; Blanca, 2015) led to vintage types. According to **Figure 5**, only one third of the variation in the ancestors was still present in the vintage accessions. Further selection and inbreeding led to the commercial varieties in the 1960s, representing only 10% of the diversity of the ancestors. However, introgressions of resistances to diseases and pests in the 1970s and 1980s increased the genetic diversity considerably, even above the level of the vintage types. The second boost of diversity, i.e., the breeding for fruit size diversity, color differences, improved taste, and additional resistances, further increased the diversity index, nearly to the level of the ancestors (**Figure 5**). We have to keep in mind here that the introgressions in the modern varieties not only descend from *S. lycopersicum* and *S. pimpinellifolium*, i.e., the species from which the domesticated tomatoes have been derived, but also from more distant relatives, including *S. peruvianum, S. pennellii, S. chilense*, and *S. habrochaites* (**Data Sheet S6**). This explains the high level of diversity of the modern varieties compared to the ancestors *S. lycopersicum* and *S. pimpinellifolium*. As the SolCap array is based on SNPs between accessions of *S. lycopersicum* and *S. pimpinellifolium* (Sim, 2012b), the genetic diversity of modern varieties harboring introgressions from other wild species might not have been fully captured by the array. It is therefore possible that the genetic diversity among the evaluated varieties from the 1970s till now might be even higher than we report.

Blanca et al. (2015) studied the domestication of tomato in Ecuador, Peru, and Mesoamerica, but also included contemporary genotypes in their analysis. These genotypes referred predominantly to genotypes from breeding programs in the USA for the fresh market and processing market. Blanca et al. concluded that genotypes for the processing and fresh market showed slightly higher levels of diversity when compared to vintage types. They mention that this is likely due to the effect of introgression during breeding, and differentiation into distinct market classes, which is consistent with our findings.

When focusing in rarefaction analyses on alleles that are present in specific (sub)groups only, so-called private alleles, Blanca et al. found that the processing contemporary types showed a higher frequency of private alleles compared to genotypes for the fresh market. The frequency of private alleles was lowest in vintage types.

Earlier work of Sim et al. (2012a) also showed that contemporary genotypes for the fresh market showed higher genetic diversity compared to the vintage sub-population. The same held for the processing group. The highest diversity was found in the cherry tomatoes, as appeared both from the genetic diversity indices as from the rarefaction analysis (Sim, 2012a).

### Suitability of the Marker Platform

The SolCap array has been based on 7,720 SNPs, discovered in RNAseq data from *S. lycopersicum* and *S. pimpinellifolium* accessions (Causse, 2002). We removed 59 markers that gave missing values for >80% of the varieties before 1970, leaving 7,661 SNP markers. These markers covered the whole tomato genome, although the number of markers per Mbp varied. After 1970, breeders started to introgress resistances from distant species, including *S. peruvianum, S. pennellii, S. chilense*, and *S. habrochaites* (**Data Sheet S6**). As the SolCap array was not designed to capture genetic variation from these species, we wondered whether the number of missing marker data would increase after 1970, due to poor hybridization of DNA sequences from these wild species to the oligos on the array. **Figure S6** shows that the number of missing data indeed increased in course of time. However, the percentage of missing marker values was still low (<​​0.5%). Therefore, we conclude that the number of missing SNP calls from the SolCap array was negligible and did not influence the conclusions.

However, we are aware that the SolCAP array may overlook quite some variation from wild species that were not included in the initial set of genotypes when selecting the SolCAP SNPs. Resequencing reveals far more SNPs, as we exemplify for two resequenced varieties (**Figure S2**). However, using, e.g., variant calling files (VCFs) based on resequencing data does have limitations too, as only reads that align to the reference genome are considered, disregarding many reads that are not aligned to the reference genome. Unmapped reads may harbor even more variation.

The selection of SNPs and their physical positions does influence the absolute values of the genetic diversity index H. Using another selection, or using resequencing data and haploblocks, rather than the SolCap array data, would influence the absolute levels of the diversity measure. However, we believe the trends will remain very comparable.

### Putting Biodiversity in a Wider Perspective Regarding Other Crops

For agricultural field crops, several studies have been performed on the genetic diversity of varieties. Wouw et al. (van de Wouw et al., 2010b) performed a meta-analysis based on data from 44 published papers, addressing diversity trends in released crop varieties of eight different field crops in the twentieth century. The studies encompassed variety diversity in many countries in the world, not only in Europe and North America. Wheat was the most represented crop, with 26 of the 44 papers focused on wheat. For wheat the lowest diversity occurred in the period from the 1960s till the 1980s. This decrease was 6% compared to the first half of the century and was significant. However, a recovery of diversity was observed in the 1990s. For seven other major crops (barley, maize, oat, flax, soybean, pea, rice), a dip in diversity was also observed in the 1960s, but the recovery was earlier compared to wheat (van de Wouw et al., 2010b). Apparently, the trends we have described for tomato are similar for other crops. However, the changes in genetic diversity have been far more pronounced and clearer for tomato, not showing an increase of a few percent, but a nine-fold increase since the 1960s. Therefore, we conclude that the concern about decreasing diversity among varieties, due to modern plant breeding, is not supported by our study. In contrast, we have observed a tremendous increase in diversity, both at the genotypic and phenotypic level.

## Data Availability Statement

Access to cultivar resequencing data can be obtained by contacting the corresponding author. All other datasets generated and analyzed for this study are included in the article/**Supplementary Material**.

## Author Contributions

HS and RV set up the initial experiments for determining biodiversity levels of varieties and made the first outlines for a paper. HS, RV, AB, YT, and YB subsequently conceptualized the paper. HS coordinated the research, performed the genetic analyses, and wrote the original draft. YT, AB, and WV were responsible for the analyses of flavor components and provided text, figures, and tables for the manuscript. YT prepared the final figures. RF did bioinformatic analyses of re-sequencing data regarding introgressions. YB analyzed introgressions of resistance genes, and RV supervised the process. All authors reviewed and rewrote (parts of) the manuscript.

## Funding

The research was financed by Plant Breeding (WUR) and by the Board for Plant Varieties, The Netherlands. The views expressed in the text are the authors’ own and do not necessarily reflect the opinions of the Dutch Board for Plant Varieties.

## Conflict of Interest

The authors declare that the research was conducted in the absence of any commercial or financial relationships that could be construed as a potential conflict of interest.

## Supplementary Material

Figure S1 The percentage of studied tomato varieties, being hybrids. The horizontal axis shows the decade of commercial introduction.

Figure S2 The frequency of SNPs, when aligning 150 bp reads of the modern cv. ‘Merlice’ to Chr. 9 of the reference tomato genome (cv. ‘Heinz’). The figure illustrates the large introgression (yellow) harboring the gene *Tm-2* *^2^* (orange) from *S. peruvianum* P.I.18650. This gene provides resistance to tomato mosaic virus (ToMV). The distal parts of Chr. 9 that show high sequence similarity to the reference genome are represented by green blocks. Possible rearrangements of the introgression compared to the reference genome are not shown.

Figure S3 The percentage of investigated tomato varieties being resistant to tomato mosaic virus.

Figure S4 The average fruit size class and weight of 284 tomato varieties (including the 90 varieties evaluated in the present study) registered in the official Dutch cultivar registry (https://www.raadvoorplantenrassen.nl/nl/rassenregister/) from 1950 till 2016. **(A)** Average fruit weight of 90 tomato varieties determined experimentally in the present study; **(B)** Relationship between fruit size class (as assigned in the official variety registry) of the 90 studied varieties and the average fruit weight determined experimentally in the present study; **(C)** Distribution of fruit sizes and calculated fruit weight of all 284 commercial varieties registered in the period from 1950 till 2016, which is divided into three sub-periods to have a balanced number of cultivars per sub-period.

Figure S5 Quantitative variation of aroma related volatile compounds in ripe fruits of 90 tomato varieties from 1950 till 2016. The five biosynthetic groups are highlighted by different colors: green – branched-chain and sulphur-containing amino acid derived volatiles, red – phenolic volatiles, blue – phenylpropanoid volatiles, yellow – lipid derived volatiles; purple – carotenoid derived volatiles. The vertical axis represents relative abundance of a compound, where the maximum abundance of a compound is set at 1.00. Each circle represents a tomato variety, and a size of a circle is proportional to the average fruit weight of that variety.

Figure S6 The proportion of SolCap SNP markers that had missing values. We took the 7661 markers into account that we used for calculation of the Nei-index *H* (Fig. 1), disregarding the markers that failed for all varieties, or failed for >80% of the varieties before 1970.

Data Sheet S1 The SolCap array SNP-scores of the 90 tomato varieties.

Data Sheet S2 Alignment of the *TM-2* and *TM-2* *^2^* alleles for resistance to the Tomato Mosaic Virus (ToMV), the susceptible allele *tm-2*, and the sequence of cv. ‘Merlice’ at this locus. This alignment shows that ‘Merlice’ harbors *TM-2* *^2^* homozygously.

Data Sheet S3 The introgressions in 90 tomato varieties, commercially introduced since 1950. These varieties were genotyped, using the SolCap array (https://www.illumina.com/library-prep-array-kit-selector/kits-and-arrays/the-solcap-tomato-consortium.html ). Column 1 to 3 provide the ColCap marker names and physical positions. A cell is blanc in case the marker scores for both alleles are identical to the prevalent score for that marker in the 1950s and 1960s. A cell is light blue, if only one marker score deviates from the ‘standard tomato genome’, therefore reflecting heterozygous introgressions. The dark blue cells represent homozygous introgressions.

Data Sheet S4 Disease resistances of the 90 varieties according the official tomato variety registration of NAKTuinbouw.

Data Sheet S5 GC-MS data of 66 annotated volatile compounds. Quantitative values of volatiles presented as counts (AU - arbitrary units) of corresponding selective ions.

Data Sheet S6 Reported resistance genes in tomato, and their positions on the tomato genome.

Data Sheet S7 Flavor components of the investigated tomato varieties. Sugar: estimated as refraction (°Brix); % juice: the amount of juice, squeezed from the pericarp relatively to the weight of the pericarp before squeezing; Acidity: the amount of titratable acid (mmol H3O+/100gr); Firmness: the power needed to break the pericarp (N). For some varieties, there were not sufficient numbers of fruits available for reliable measurements.

Table S1 List of studied tomato varieties. The varieties are sorted according to their years of commercial introduction in The Netherlands according to the Dutch Variety Register of the Raad voor Plantenrassen (https://nederlandsrassenregister.nl/).

Table S2 Trends for basic flavor parameters. Mann-Kendall trend statistics for all the varieties studied (*n* = 90), and for the non-cherry (medium-sized and large fruits, >30 g/fruit) varieties. Trend coefficient *S* estimates strength and directionality of a trend. The statistical significance is represented by a P-value. Trends are estimated for the entire collection of 90 varieties, and for a sub-set of medium-sized and large fruited varieties (Non-cherry). SSC – soluble solid content, equivalent of sugar content in tomato, TA – titratable acidity, Firmness – firmness of fruit pericarp, Juiciness – amount of liquid released from tomato fruit pericarp.

Table S3 Volatile compounds which had a statistically significant quantitative trends in fruits of tomato varieties introduced from 1950 till 2016. Directionality and strength of trends is represented by Mann-Kendall trend coefficient S with positive and negative values indicating an increase and decrease, respectivelly, in concentration during the period of time studied. Trends are estimated in the entire collection of 90 varieties and in a sub-set of medium and large fruited varieties (Non-cherry). P-values represent significance of the trend
