# Supplementary material for: Breeding Has Increased the Diversity of Cultivated Tomato in The Netherlands
Source: Front Plant Sci. 2019 Dec 20;10:1606. doi: 10.3389/fpls.2019.01606 (PMC6932954; doi:10.3389/fpls.2019.01606)
Supplement: Figure S1 — The percentage of studied tomato varieties, being hybrids. The horizontal axis shows the decade of commercial introduction. [file Image_1.pdf]

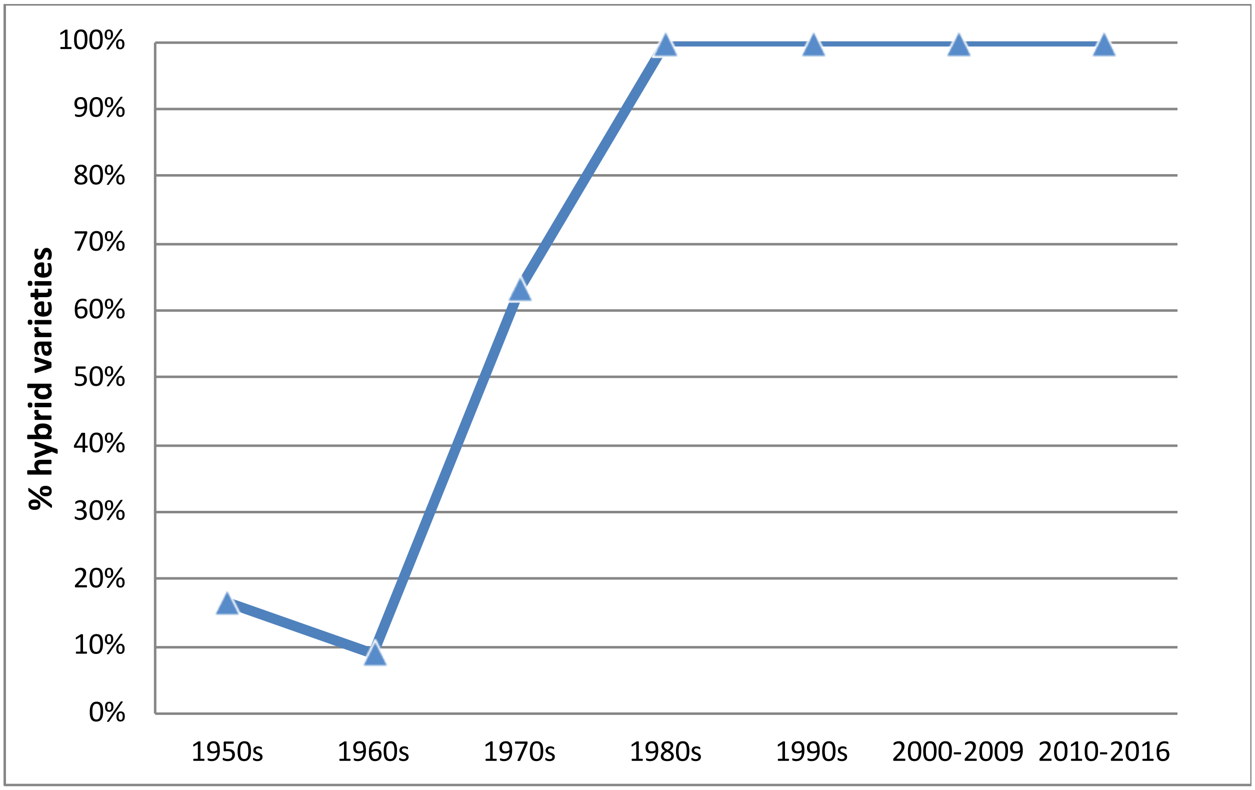

Fig. S1. The percentage of studied tomato varieties, being hybrids. The horizontal axis shows the decade of commercial introduction.
