# Supplementary material for: Breeding Has Increased the Diversity of Cultivated Tomato in The Netherlands
Source: Front Plant Sci. 2019 Dec 20;10:1606. doi: 10.3389/fpls.2019.01606 (PMC6932954; doi:10.3389/fpls.2019.01606)
Supplement: Figure S2 — The frequency of SNPs, when aligning 150 bp reads of the modern cv. ‘Merlice’ to Chr. 9 of the reference tomato genome (cv. ‘Heinz’). The figure illustrates the large introgression (yellow) harboring the gene Tm-2 2 (orange) from S. peruvianum P.I.18650. This gene provides resistance to tomato mosaic virus (ToMV). The distal parts of Chr. 9 that show high sequence similarity to the reference genome are represented by green blocks. Possible rearrangements of the introgression compared to the reference genome are not shown. [file Image_2.pdf]

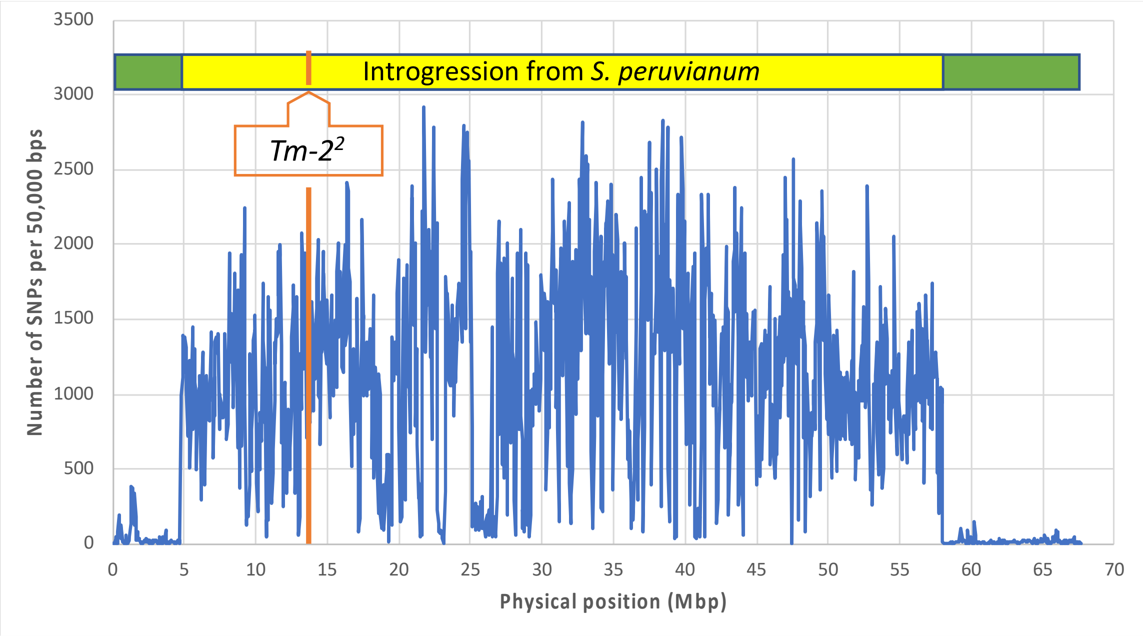

Fig. S2. The frequency of SNPs, when aligning 150 bp reads of the modern cv. 'Merlice' to Chr. 9 of the reference tomato genome (cv. 'Heinz'). The figure illustrates the large introgression (yellow) harboring the gene *Tm-2*^{2} (orange) from *S. peruvianum* P.I.18650. This gene provides resistance to tomato mosaic virus (ToMV). The distal parts of Chr. 9 that show high sequence similarity to the reference genome are represented by green blocks. Possible rearrangements of the introgression compared to the reference genome are not shown.
