# Supplementary figures and images for: Breeding Has Increased the Diversity of Cultivated Tomato in The Netherlands
Source: Front Plant Sci. 2019 Dec 20;10:1606. doi: 10.3389/fpls.2019.01606 (PMC6932954; doi:10.3389/fpls.2019.01606)

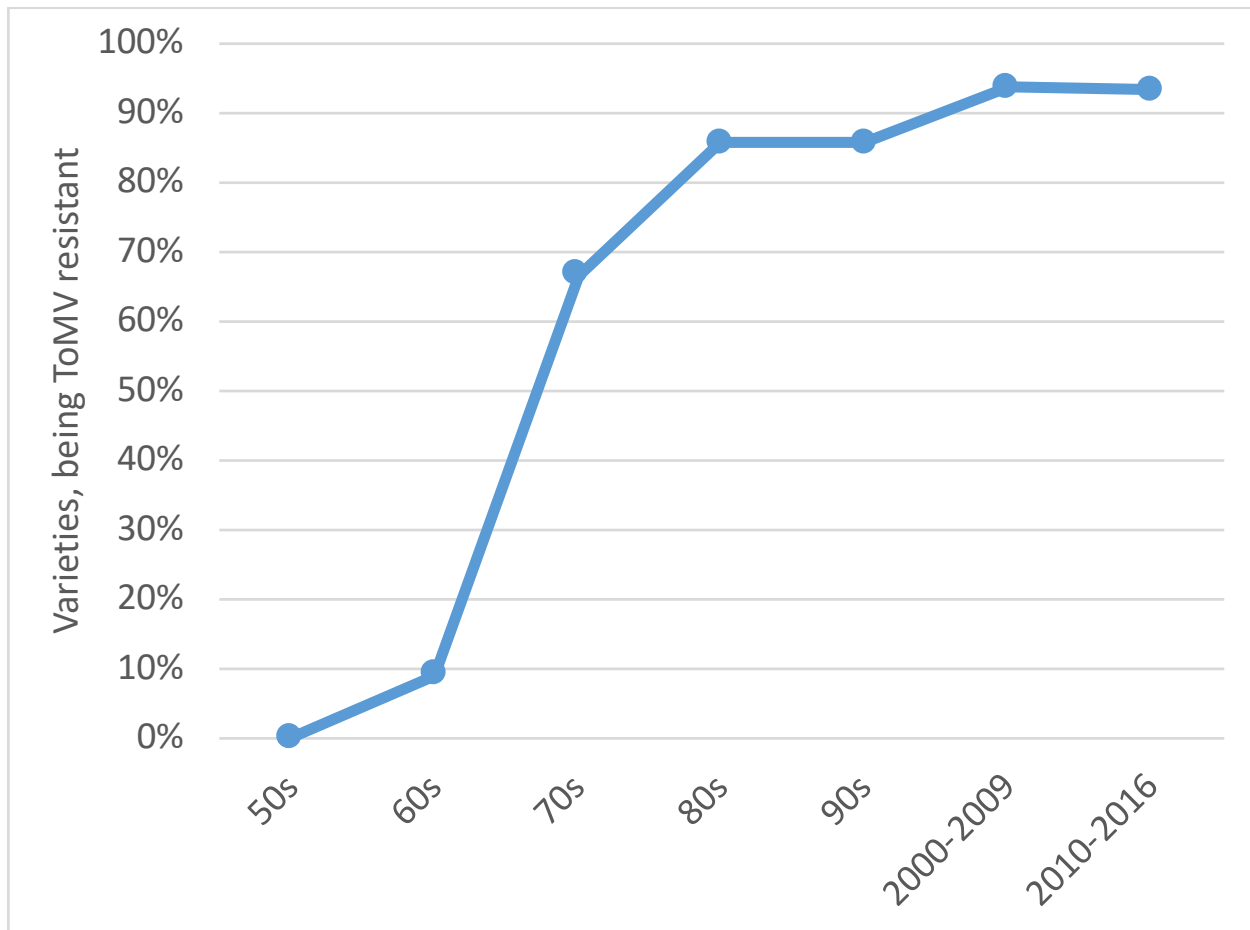

**Fig. S3.** The percentage of investigated tomato varieties being resistant to tomato mosaic virus.

Supplement: Figure S3 — The percentage of investigated tomato varieties being resistant to tomato mosaic virus. [file Image_3.pdf]
