# Supplementary material for: Breeding Has Increased the Diversity of Cultivated Tomato in The Netherlands
Source: Front Plant Sci. 2019 Dec 20;10:1606. doi: 10.3389/fpls.2019.01606 (PMC6932954; doi:10.3389/fpls.2019.01606)
Supplement: Figure S4 — The average fruit size class and weight of 284 tomato varieties (including the 90 varieties evaluated in the present study) registered in the official Dutch cultivar registry (https://www.raadvoorplantenrassen.nl/nl/rassenregister/) from 1950 till 2016. (A) Average fruit weight of 90 tomato varieties determined experimentally in the present study; (B) Relationship between fruit size class (as assigned in the official variety registry) of the 90 studied varieties and the average fruit weight determined experimentally in the present study; (C) Distribution of fruit sizes and calculated fruit weight of all 284 commercial varieties registered in the period from 1950 till 2016, which is divided into three sub-periods to have a balanced number of cultivars per sub-period. [file Image_4.pdf]

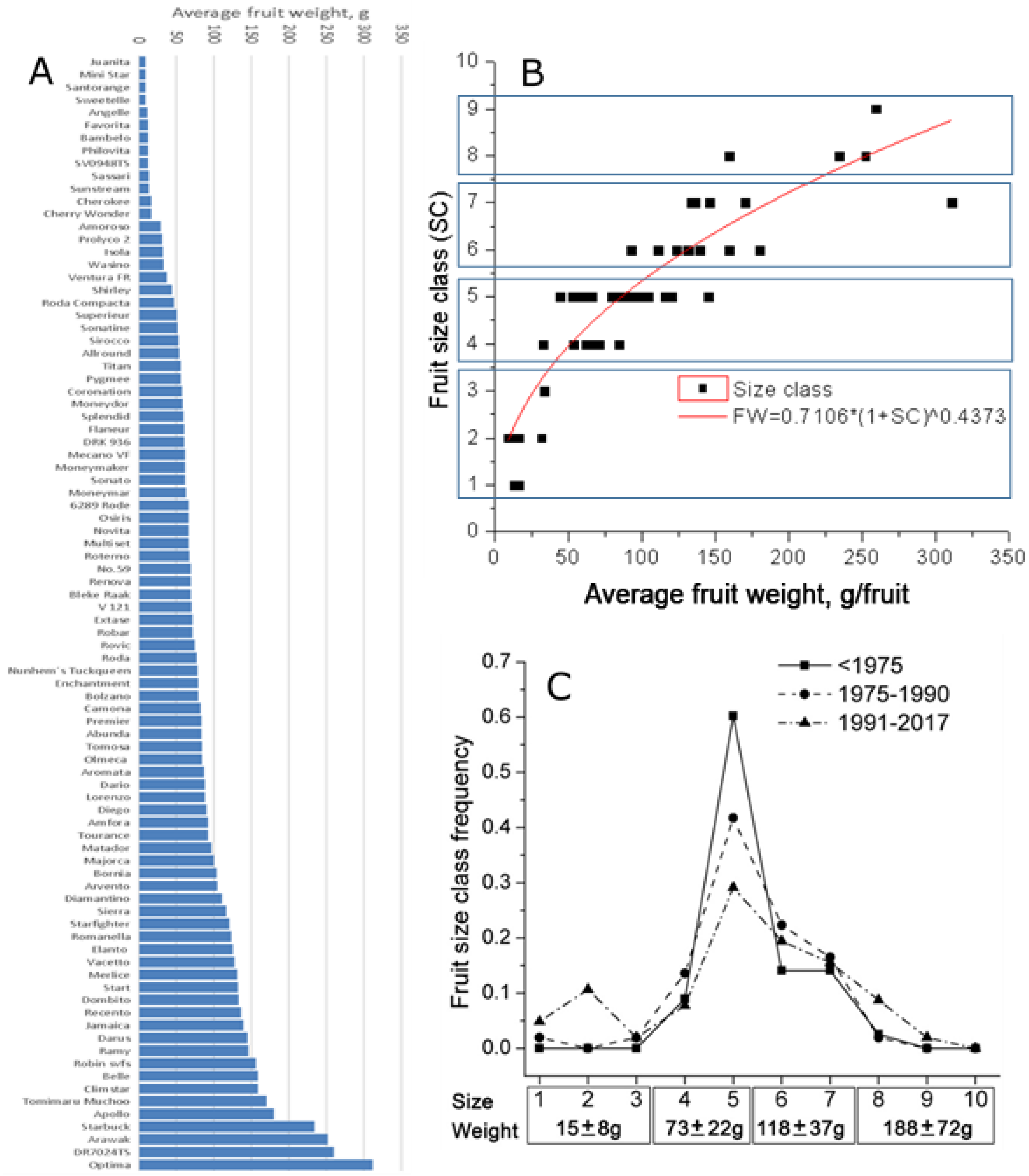

Fig. S4. The average fruit size class and weight of 284 tomato varieties (including the 90 varieties evaluated in the present study) registered in the official Dutch cultivar registry (https://www.raadvoorplantenrassen.nl/nl/rassenregister/) from 1950 till 2016. (**A)** Average fruit weight of 90 tomato varieties determined experimentally in the present study; (**B**) Relationship between fruit size class (as assigned in the official variety registry) of the 90 studied varieties and the average fruit weight determined experimentally in the present study; (**C**) Distribution of fruit sizes and calculated fruit weight of all 284 commercial varieties registered in the period from 1950 till 2016, which is divided into three sub-periods to have a balanced number of cultivars per sub-period.
