# Supplementary material for: Breeding Has Increased the Diversity of Cultivated Tomato in The Netherlands
Source: Front Plant Sci. 2019 Dec 20;10:1606. doi: 10.3389/fpls.2019.01606 (PMC6932954; doi:10.3389/fpls.2019.01606)
Supplement: Figure S5 — Quantitative variation of aroma related volatile compounds in ripe fruits of 90 tomato varieties from 1950 till 2016. The five biosynthetic groups are highlighted by different colors: green – branched-chain and sulphur-containing amino acid derived volatiles, red – phenolic volatiles, blue – phenylpropanoid volatiles, yellow – lipid derived volatiles; purple – carotenoid derived volatiles. The vertical axis represents relative abundance of a compound, where the maximum abundance of a compound is set at 1.00. Each circle represents a tomato variety, and a size of a circle is proportional to the average fruit weight of that variety. [file Image_5.pdf]

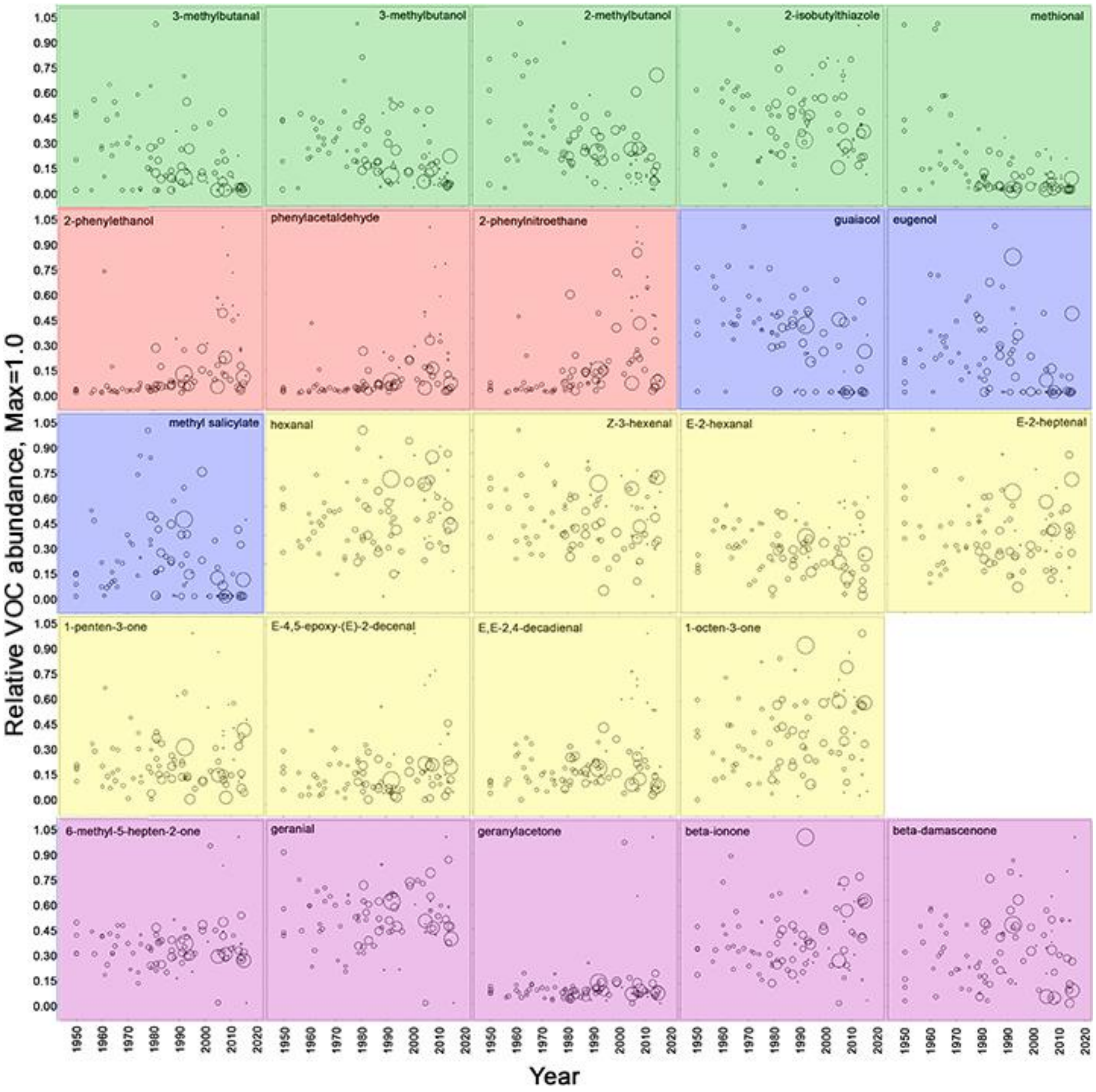

Fig. S5. Quantitative variation of aroma related volatile compounds in ripe fruits of 90 tomato varieties from 1950 till 2016. The five biosynthetic groups are highlighted by different colors: green – branched-chain and sulphur-containing amino acid derived volatiles, red – phenolic volatiles, blue – phenylpropanoid volatiles, yellow – lipid derived volatiles; purple – carotenoid derived volatiles. The vertical axis represents relative abundance of a compound, where the maximum abundance of a compound is set at 1.00. Each circle represents a tomato variety, and a size of a circle is proportional to the average fruit weight of that variety.
