# Supplementary material for: Breeding Has Increased the Diversity of Cultivated Tomato in The Netherlands
Source: Front Plant Sci. 2019 Dec 20;10:1606. doi: 10.3389/fpls.2019.01606 (PMC6932954; doi:10.3389/fpls.2019.01606)
Supplement: Figure S6 — The proportion of SolCap SNP markers that had missing values. We took the 7661 markers into account that we used for calculation of the Nei-index H (Fig. 1), disregarding the markers that failed for all varieties, or failed for >80% of the varieties before 1970. [file Image_6.pdf]

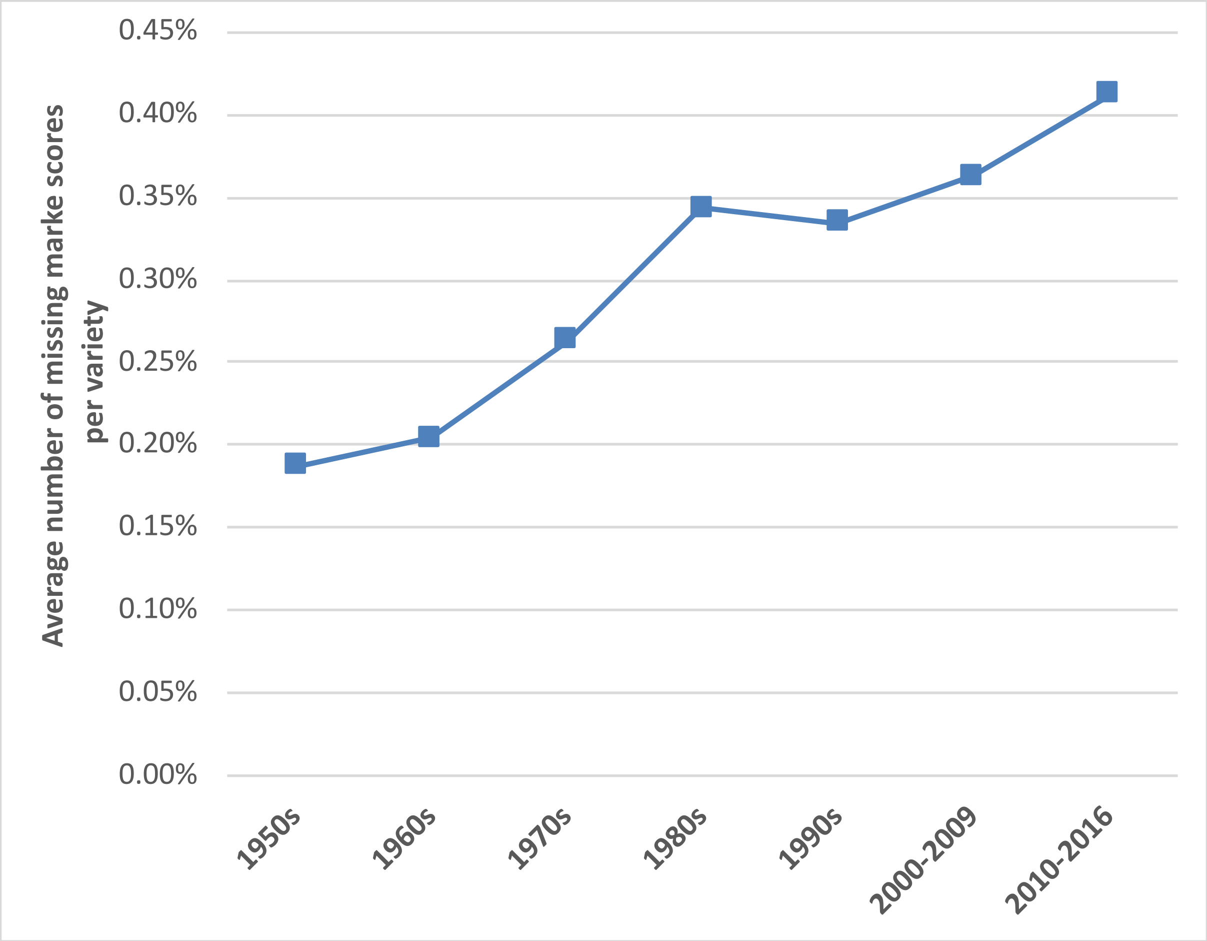

Fig. S6. The proportion of SolCap SNP markers that had missing values. We took the 7661 markers into account that we used for calculation of the Nei-index *H* (Fig. 1), disregarding the markers that failed for all varieties, or failed for >80% of the varieties before 1970.
