# Supplementary material for: Breeding Has Increased the Diversity of Cultivated Tomato in The Netherlands
Source: Front Plant Sci. 2019 Dec 20;10:1606. doi: 10.3389/fpls.2019.01606 (PMC6932954; doi:10.3389/fpls.2019.01606)
Supplement: Data Sheet S2 — Alignment of the TM-2 and TM-2 2 alleles for resistance to the Tomato Mosaic Virus (ToMV), the susceptible allele tm-2, and the sequence of cv. ‘Merlice’ at this locus. This alignment shows that ‘Merlice’ harbors TM-2 2 homozygously. [file DataSheet_2.pdf]

Data S2. Alignment of the TM-2 and TM-2[2] alleles for resistance to the Tomato Mosaic Virus (ToMV), the susceptible allele tm-2, and the sequence of cv. 'Merlice' at this locus. This alignment shows that 'Merlice' harbors TM-2[2] homozygously.

Article title: Breeding has increased the diversity of cultivated tomato
Authors: Henk J. Schouten, Yury Tikunov, Wouter Verkerke, Richard Finkers, Arnaud Bovy, Yuling Bai, Richard G.F. Visser

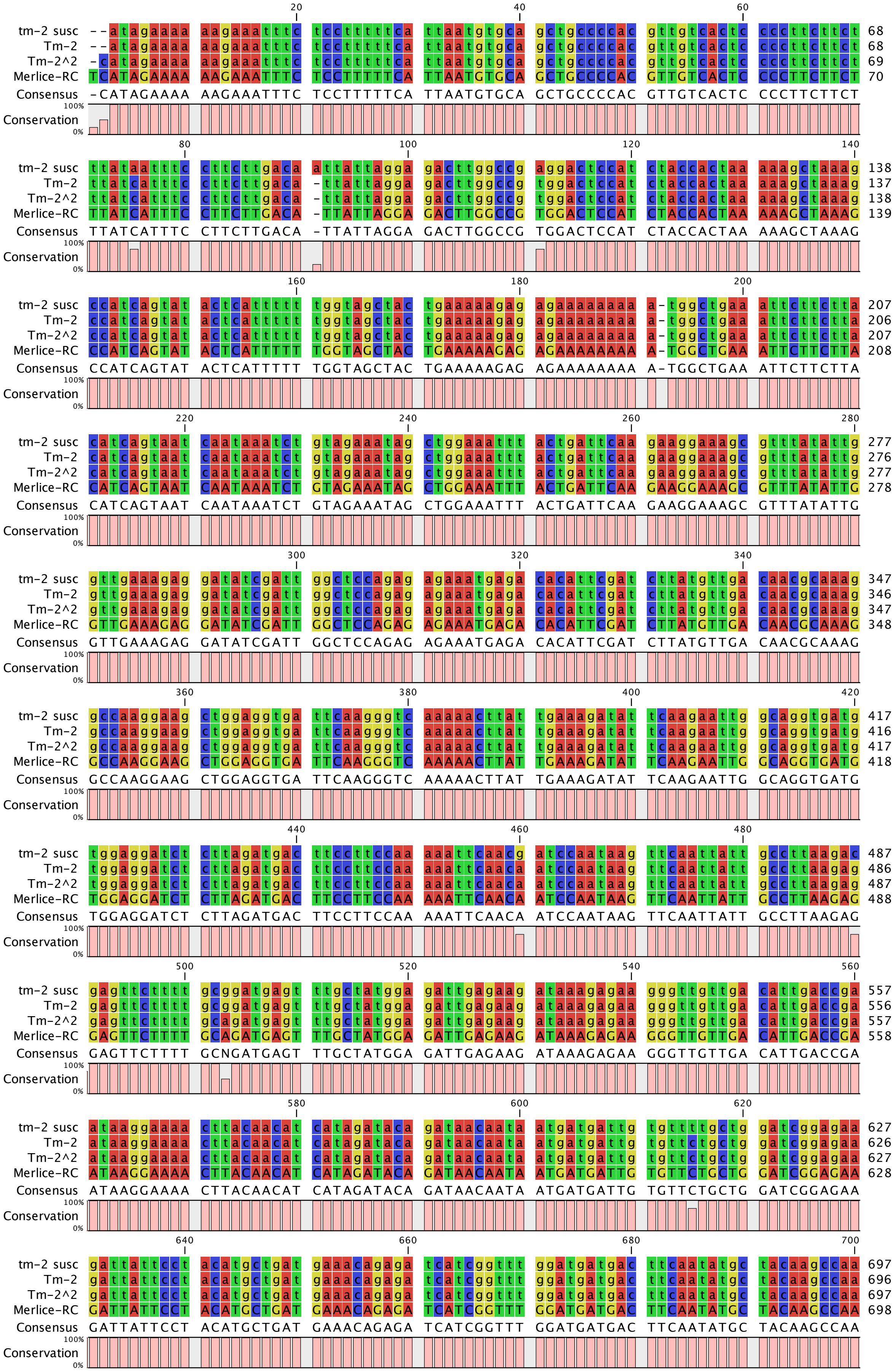
20 40 60
tm-2 susc --atagaaaa aagaaatttc tcctttttca ttaatgtgca gctgccccac gttgtcactc cccttcttct 68
Tm-2 --atagaaaa aagaaatttc tcctttttca ttaatgtgca gctgccccac gttgtcactc cccttcttct 68
Tm-2^2 -catagaaaa aagaaatttc tcctttttca ttaatgtgca gctgccccac gttgtcactc cccttcttct 69
Merlice-RC TCATAGAAAA AAGAAATTTC TCCTTTTTCA TTAATGTGCA GCTGCCCCAC GTTGTCACTC CCCTTCTTCT 70
Consensus -CATAGAAAA AAGAAATTTC TCCTTTTTCA TTAATGTGCA GCTGCCCCAC GTTGTCACTC CCCTTCTTCT
Conservation 100% 0%
80 100 120 140
tm-2 susc ttataatttc cttcttgaca attattagga gacttggccg aggactccat ctaccactaa aaagctaaag 138
Tm-2 ttatcatttc cttcttgaca -ttattagga gacttggccg tggactccat ctaccactaa aaagctaaag 137
Tm-2^2 ttatcatttc cttcttgaca -ttattagga gacttggccg tggactccat ctaccactaa aaagctaaag 138
Merlice-RC TTATCATTTC CTTCTTGACA -TTATTAGGA GACTTGGCCG TGGACTCCAT CTACCACTAA AAAGCTAAAG 139
Consensus TTATCATTTC CTTCTTGACA -TTATTAGGA GACTTGGCCG TGGACTCCAT CTACCACTAA AAAGCTAAAG
Conservation 100% 0%
160 180 200
tm-2 susc ccatcagtat actcattttt tggtagctac tgaaaaagag agaaaaaaaa a-tggctgaa attcttctta 207
Tm-2 ccatcagtat actcattttt tggtagctac tgaaaaagag agaaaaaaaa a-tggctgaa attcttctta 206
Tm-2^2 ccatcagtat actcattttt tggtagctac tgaaaaagag agaaaaaaaa a-tggctgaa attcttctta 207
Merlice-RC CCATCAGTAT ACTCATTTTT TGGTAGCTAC TGAAAAAGAG AGAAAAAAAA A-TGGCTGAA ATTCTTCTTA 208
Consensus CCATCAGTAT ACTCATTTTT TGGTAGCTAC TGAAAAAGAG AGAAAAAAAA A-TGGCTGAA ATTCTTCTTA
Conservation 100% 0%
220 240 260 280
tm-2 susc catcagtaat caataaatct gtagaaatag ctggaaattt actgattcaa gaaggaaagc gtttatattg 277
Tm-2 catcagtaat caataaatct gtagaaatag ctggaaattt actgattcaa gaaggaaagc gtttatattg 276
Tm-2^2 catcagtaat caataaatct gtagaaatag ctggaaattt actgattcaa gaaggaaagc gtttatattg 277
Merlice-RC CATCAGTAAT CAATAAATCT GTAGAAATAG CTGGAAATTT ACTGATTCAA GAAGGAAAGC GTTTATATTG 278
Consensus CATCAGTAAT CAATAAATCT GTAGAAATAG CTGGAAATTT ACTGATTCAA GAAGGAAAGC GTTTATATTG
Conservation 100% 0%
300 320 340
tm-2 susc gttgaaagag gatatcgatt ggctccagag agaaatgaga cacattcgat cttatgttga caacgcaaag 347
Tm-2 gttgaaagag gatatcgatt ggctccagag agaaatgaga cacattcgat cttatgttga caacgcaaag 346
Tm-2^2 gttgaaagag gatatcgatt ggctccagag agaaatgaga cacattcgat cttatgttga caacgcaaag 347
Merlice-RC GTTGAAAGAG GATATCGATT GGCTCCAGAG AGAAATGAGA CACATTCGAT CTTATGTTGA CAACGCAAAG 348
Consensus GTTGAAAGAG GATATCGATT GGCTCCAGAG AGAAATGAGA CACATTCGAT CTTATGTTGA CAACGCAAAG
Conservation 100% 0%
360 380 400 420
tm-2 susc gccaaggaag ctggaggtga ttcaagggtc aaaaacttat tgaaagatat tcaagaattg gcaggtgatg 417
Tm-2 gccaaggaag ctggaggtga ttcaagggtc aaaaacttat tgaaagatat tcaagaattg gcaggtgatg 416
Tm-2^2 gccaaggaag ctggaggtga ttcaagggtc aaaaacttat tgaaagatat tcaagaattg gcaggtgatg 417
Merlice-RC GCCAAGGAAG CTGGAGGTGA TTCAAGGGTC AAAAACTTAT TGAAAGATAT TCAAGAATTG GCAGGTGATG 418
Consensus GCCAAGGAAG CTGGAGGTGA TTCAAGGGTC AAAAACTTAT TGAAAGATAT TCAAGAATTG GCAGGTGATG
Conservation 100% 0%
440 460 480
tm-2 susc tggaggatct cttagatgac ttccttccaa aaattcaacg atccaataag ttcaattatt gccttaagac 487
Tm-2 tggaggatct cttagatgac ttccttccaa aaattcaaca atccaataag ttcaattatt gccttaagag 486
Tm-2^2 tggaggatct cttagatgac ttccttccaa aaattcaaca atccaataag ttcaattatt gccttaagag 487
Merlice-RC TGGAGGATCT CTTAGATGAC TTCCTTCCAA AAATTCAACA ATCCAATAAG TTCAATTATT GCCTTAAGAG 488
Consensus TGGAGGATCT CTTAGATGAC TTCCTTCCAA AAATTCAACA ATCCAATAAG TTCAATTATT GCCTTAAGAG
Conservation 100% 0%
500 520 540 560
tm-2 susc gagttctttt gcggatgagt ttgctatgga gattgagaag ataaagagaa gggttgttga cattgaccga 557
Tm-2 gagttctttt gcggatgagt ttgctatgga gattgagaag ataaagagaa gggttgttga cattgaccga 556
Tm-2^2 gagttctttt gcagatgagt ttgctatgga gattgagaag ataaagagaa gggttgttga cattgaccga 557
Merlice-RC GAGTTCTTTT GCAGATGAGT TTGCTATGGA GATTGAGAAG ATAAAGAGAA GGGTTGTTGA CATTGACCGA 558
Consensus GAGTTCTTTT GCNGATGAGT TTGCTATGGA GATTGAGAAG ATAAAGAGAA GGGTTGTTGA CATTGACCGA
Conservation 100% 0%
580 600 620
tm-2 susc ataaggaaaa cttacaacat catagataca gataacaata atgatgattg tgttttgctg gatcggagaa 627
Tm-2 ataaggaaaa cttacaacat catagataca gataacaata atgatgattg tgttctgctg gatcggagaa 626
Tm-2^2 ataaggaaaa cttacaacat catagataca gataacaata atgatgattg tgttctgctg gatcggagaa 627
Merlice-RC ATAAGGAAAA CTTACAACAT CATAGATACA GATAACAATA ATGATGATTG TGTTCTGCTG GATCGGAGAA 628
Consensus ATAAGGAAAA CTTACAACAT CATAGATACA GATAACAATA ATGATGATTG TGTTCTGCTG GATCGGAGAA
Conservation 100% 0%
640 660 680 700
tm-2 susc gattattcct acatgctgat gaaacagaga tcatcggttt ggatgatgac ttcaatatgc tacaagccaa 697
Tm-2 gattattcct acatgctgat gaaacagaga tcatcggttt ggatgatgac ttcaatatgc tacaagccaa 696
Tm-2^2 gattattcct acatgctgat gaaacagaga tcatcggttt ggatgatgac ttcaatatgc tacaagccaa 697
Merlice-RC GATTATTCCT ACATGCTGAT GAAACAGAGA TCATCGGTTT GGATGATGAC TTCAATATGC TACAAGCCAA 698
Consensus GATTATTCCT ACATGCTGAT GAAACAGAGA TCATCGGTTT GGATGATGAC TTCAATATGC TACAAGCCAA
Conservation 100% 0%

```
                       720                   740                   760
tm-2 susc   attactcaat caagatttgc attatggagt tgtttccata gttggcatgc ccggtctggg gaaaacaact   767
Tm-2        attacttaat caagatttgc attatggagt tgtttccata gttggcatgc ccggtctggg gaaaacaact   766
Tm-2^2      attacttaat caagatttgc attatggagt tgtttccata gttggcatgc ccggtctggg gaaaacaact   767
Merlice-RC  ATTACTTAAT CAAGATTTGC ATTATGGAGT TGTTTCCATA GTTGGCATGC CCGGTCTGGG GAAAACAACT   768
Consensus   ATTACTTAAT CAAGATTTGC ATTATGGAGT TGTTTCCATA GTTGGCATGC CCGGTCTGGG GAAAACAACT
Conservation (100% / 0%)

            780                   800                   820                   840
tm-2 susc   cttgccaaga aactttatag gctcattcgt gatcaatttg agtgttctgg actggtctac gtttcacaac   837
Tm-2        cttgccaaga aactttatag gctcattcgt gatcaatttg agtgttctgg actggtctac gtttcacaac   836
Tm-2^2      cttgccaaga aactttatag gctcattcgt gatcaatttg agtgttctgg actggtctac gtttcacaac   837
Merlice-RC  CTTGCCAAGA AACTTTATAG GCTCATTCGT GATCAATTTG AGTGTTCTGG ACTGGTCTAC GTTTCACAAC   838
Consensus   CTTGCCAAGA AACTTTATAG GCTCATTCGT GATCAATTTG AGTGTTCTGG ACTGGTCTAC GTTTCACAAC
Conservation (100% / 0%)

                       860                   880                   900
tm-2 susc   agccaagagc gggtgaaatc ttacttgaca ttgccaaaca aattggactg acggaacaga aaattaagga   907
Tm-2        agccaagagc gagtgaaatc ttacttgaca ttgccaaaca aattggactg acggaacaga aaatgaagga   906
Tm-2^2      agccaagagc gagtgaaatc ttacttgaca ttgccaaaca aattggactg acggaacaga aaatgaagga   907
Merlice-RC  AGCCAAGAGC GAGTGAAATC TTACTTGACA TTGCCAAACA AATTGGACTG ACGGAACAGA AAATGAAGGA   908
Consensus   AGCCAAGAGC GAGTGAAATC TTACTTGACA TTGCCAAACA AATTGGACTG ACGGAACAGA AAATGAAGGA
Conservation (100% / 0%)

            920                   940                   960                   980
tm-2 susc   aaatttggag gacaacctgc gatcactctt gaaaataaaa aggtatgtta tcctcctaga tgacatttgg   977
Tm-2        aaatttggag gacaacctgc gatcactctt gaaaataaaa aggtatgttt tcctcctaga tgacatttgg   976
Tm-2^2      aaatttggag gacaacctgc gatcactctt gaaaataaaa aggtatgtta tcctcctaga tgacatttgg   977
Merlice-RC  AAATTTGGAG GACAACCTGC GATCACTCTT GAAAATAAAA AGGTATGTTA TCCTCCTAGA TGACATTTGG   978
Consensus   AAATTTGGAG GACAACCTGC GATCACTCTT GAAAATAAAA AGGTATGTTA TCCTCCTAGA TGACATTTGG
Conservation (100% / 0%)

                     1,000                 1,020                 1,040
tm-2 susc   gatgttgaaa tttgggatga tctgaaactt gtccttcctg aatgtgactc aaaagtcggc agtagaatga   1047
Tm-2        gatgtggaaa tttgggatga tctgaaactt gtccttcctg aatgtgattc aaaagtcggc agtagaatta   1046
Tm-2^2      gatgtggaaa tttgggatga tctgaaactt gtccttcctg aatgtgattc aaaagtcggc agtagaatga   1047
Merlice-RC  GATGTGGAAA TTTGGGATGA TCTGAAACTT GTCCTTCCTG AATGTGATTC AAAAGTCGGC AGTAGAATGA   1048
Consensus   GATGTGGAAA TTTGGGATGA TCTGAAACTT GTCCTTCCTG AATGTGATTC AAAAGTCGGC AGTAGAATGA
Conservation (100% / 0%)

          1,060                 1,080                 1,100                 1,120
tm-2 susc   taatcacgtc tcgaaatagt aatgtaggca gatacatagg aggggaatcc tccctccatg cattgcaacc   1117
Tm-2        taatcacgtc tcgaaatagt aatgtaggca gatacatagg aggggaatcc tccctccatg cattgcaacc   1116
Tm-2^2      taatcacgtc tcgaaatagt aatgtaggca gatacatagg aggggaatcc tccctccatg cattgcaacc   1117
Merlice-RC  TAATCACGTC TCGAAATAGT AATGTAGGCA GATACATAGG AGGGGAATCC TCCCTCCATG CATTGCAACC   1118
Consensus   TAATCACGTC TCGAAATAGT AATGTAGGCA GATACATAGG AGGGGAATCC TCCCTCCATG CATTGCAACC
Conservation (100% / 0%)

                     1,140                 1,160                 1,180
tm-2 susc   cctagaatcc gagaaaagct ttgaactctt taccaagaaa atctttaatt ttgatgataa taatagttgg   1187
Tm-2        cctagaatcc gagaaaagct ttgaactctt taccaagaaa atctttaatt ttgatgataa taatagttgg   1186
Tm-2^2      cctagaatcc gagaaaagct ttgaactctt taccaagaaa atctttaatt ttgatgataa taatagttgg   1187
Merlice-RC  CCTAGAATCC GAGAAAAGCT TTGAACTCTT TACCAAGAAA ATCTTTAATT TTGATGATAA TAATAGTTGG   1188
Consensus   CCTAGAATCC GAGAAAAGCT TTGAACTCTT TACCAAGAAA ATCTTTAATT TTGATGATAA TAATAGTTGG
Conservation (100% / 0%)

          1,200                 1,220                 1,240                 1,260
tm-2 susc   gccaatgctt cacctgactt ggtgaatatt ggtagaaata tagctgggag atgtggaggt ataccgctag   1257
Tm-2        gccaatgctt cacctgactt ggtgaatatt ggtagaaata tagttgggag atgtggaggt ataccgctag   1256
Tm-2^2      gccaatgctt cacctgactt ggtgaatatt ggtagaaata tagttgggag atgtggaggt ataccgctag   1257
Merlice-RC  GCCAATGCTT CACCTGACTT GGTGAATATT GGTAGAAATA TAGTTGGGAG ATGTGGAGGT ATACCGCTAG   1258
Consensus   GCCAATGCTT CACCTGACTT GGTGAATATT GGTAGAAATA TAGTTGGGAG ATGTGGAGGT ATACCGCTAG
Conservation (100% / 0%)

                     1,280                 1,300                 1,320
tm-2 susc   ccatagtggt gactgcaggc atgttaaggg caagagaaag aacagaacat gcgtggaaca gagtacttga   1327
Tm-2        ccatagtggt gactgcaggc atgttaaggg caagagaaag aacagaacat gcgtggaaca gagtacttga   1326
Tm-2^2      ccatagtggt gactgcaggc atgttaaggg caagagaaag aacagaacat gcgtggaaca gagtacttga   1327
Merlice-RC  CCATAGTGGT GACTGCAGGC ATGTTAAGGG CAAGAGAAAG AACAGAACAT GCGTGGAACA GAGTACTTGA   1328
Consensus   CCATAGTGGT GACTGCAGGC ATGTTAAGGG CAAGAGAAAG AACAGAACAT GCGTGGAACA GAGTACTTGA
Conservation (100% / 0%)

          1,340                 1,360                 1,380                 1,400
tm-2 susc   gagtatgggc cataaagttc aagatggatg tgctaaggta ttggctctca gttacaatga tttaccgatt   1397
Tm-2        gagtatgggc cataaagttc aagatggatg tgctaaggta ttggctctca gttacaatga tttacctatt   1396
Tm-2^2      gagtatgggc cataaagttc aagatggatg tgctaaggta ttggctctca gttacaatga tttacctatt   1397
Merlice-RC  GAGTATGGGC CATAAAGTTC AAGATGGATG TGCTAAGGTA TTGGCTCTCA GTTACAATGA TTTACCTATT   1398
Consensus   GAGTATGGGC CATAAAGTTC AAGATGGATG TGCTAAGGTA TTGGCTCTCA GTTACAATGA TTTACCTATT
Conservation (100% / 0%)
```

```
                                       1,420                          1,440                          1,460
tm-2 susc    gcctcaaggc  catgtttctt  gtactttagc  ctttaccccg  aggaccatga  aattcgtgct  tttgatttga  1467
Tm-2         gcctcaaggc  catgtttctt  gtactttggc  ctttaccccg  aggaccatga  aattcgtgct  tttgatttga  1466
Tm-2^2       gcctcaaggc  catgtttctt  gtactttggc  ctttaccccg  aggaccatga  aattcgtgct  tttgatttga  1467
Merlice-RC   GCCTCAAGGC  CATGTTTCTT  GTACTTTGGC  CTTTACCCCG  AGGACCATGA  AATTCGTGCT  TTTGATTTGA  1468
Consensus    GCCTCAAGGC  CATGTTTCTT  GTACTTTGGC  CTTTACCCCG  AGGACCATGA  AATTCGTGCT  TTTGATTTGA
100%
Conservation
0%
```

```
                          1,480                          1,500                          1,520                          1,540
tm-2 susc    taaatatgtg  gattgctgag  aagtttattg  tagtaaatag  tggtaatagg  cgagaggctg  aggatttggc  1537
Tm-2         taaatatgtg  gattgctgag  aagtttatag  tagtaaatag  tggtaatagg  cgagaggctg  aggatttggc  1536
Tm-2^2       taaatatgtg  gattgctgag  aagtttatag  tagtaaatag  tggtaatagg  cgagaggctg  aggatttggc  1537
Merlice-RC   TAAATATGTG  GATTGCTGAG  AAGTTTATAG  TAGTAAATAG  TGGTAATAGG  CGAGAGGCTG  AGGATTTGGC  1538
Consensus    TAAATATGTG  GATTGCTGAG  AAGTTTATAG  TAGTAAATAG  TGGTAATAGG  CGAGAGGCTG  AGGATTTGGC
100%
Conservation
0%
```

```
                                       1,560                          1,580                          1,600
tm-2 susc    ggaggacgtc  ctaaatgatt  tggtttctag  aaacttgatt  caacttgcca  aaaggacata  taatggaaga  1607
Tm-2         ggaggacgtc  ctaaatgatt  tggtttctag  aaacttgatt  caacttgcca  aaaggacata  taatggaaga  1606
Tm-2^2       ggaggacgtc  ctaaatgatt  tggtttctag  aaacttgatt  caacttgcca  aaaggacata  taatggaaga  1607
Merlice-RC   GGAGGACGTC  CTAAATGATT  TGGTTTCTAG  AAACTTGATT  CAACTTGCCA  AAAGGACATA  TAATGGAAGA  1608
Consensus    GGAGGACGTC  CTAAATGATT  TGGTTTCTAG  AAACTTGATT  CAACTTGCCA  AAAGGACATA  TAATGGAAGA
100%
Conservation
0%
```

```
                          1,620                          1,640                          1,660                          1,680
tm-2 susc    atttcaagtt  gtcgcataca  tgacttgtta  catagtttgt  gtgtggactt  ggctaaggaa  agtaacttct  1677
Tm-2         atttcaagtt  gtcgcataca  tgacttgtta  catagtttgt  gtgtggactt  ggctaaggaa  agtaacttct  1676
Tm-2^2       atttcaagtt  gtcgcataca  tgacttgtta  catagtttgt  gtgtggactt  ggctaaggaa  agtaacttct  1677
Merlice-RC   ATTTCAAGTT  GTCGCATACA  TGACTTGTTA  CATAGTTTGT  GTGTGGACTT  GGCTAAGGAA  AGTAACTTCT  1678
Consensus    ATTTCAAGTT  GTCGCATACA  TGACTTGTTA  CATAGTTTGT  GTGTGGACTT  GGCTAAGGAA  AGTAACTTCT
100%
Conservation
0%
```

```
                                       1,700                          1,720                          1,740
tm-2 susc    ttcacaccgc  gcatgatgta  tttggtgatc  ccggcaatgt  cgctaggctt  cgaaggatta  cattctactc  1747
Tm-2         ttcacaccgc  gcatgatgca  tttggtgatc  ccggcaatgt  tgctaggctc  cgaaggatta  cattctactc  1746
Tm-2^2       ttcacaccgc  gcatgatgca  tttggtgatc  ccggcaatgt  tgctaggctc  cgaaggatta  cattctactc  1747
Merlice-RC   TTCACACCGC  GCATGATGCA  TTTGGTGATC  CCGGCAATGT  TGCTAGGCTC  CGAAGGATTA  CATTCTACTC  1748
Consensus    TTCACACCGC  GCATGATGCA  TTTGGTGATC  CCGGCAATGT  TGCTAGGCTC  CGAAGGATTA  CATTCTACTC
100%
Conservation
0%
```

```
                          1,760                          1,780                          1,800                          1,820
tm-2 susc    tgacaatgtc  atgattgagt  tcttcggttc  taatcctaag  cttgagaagc  ttcgtgtact  tttctgtttc  1817
Tm-2         tgacaatgtc  atgattgagt  tcttccgttc  aaatcctaag  cttgagaagc  ttcgtgtact  tttctgtttc  1816
Tm-2^2       tgacaatgtc  atgattgagt  tcttccgttc  aaatcctaag  cttgagaagc  ttcgtgtact  tttctgtttc  1817
Merlice-RC   TGACAATGTC  ATGATTGAGT  TCTTCCGTTC  AAATCCTAAG  CTTGAGAAGC  TTCGTGTACT  TTTCTGTTTC  1818
Consensus    TGACAATGTC  ATGATTGAGT  TCTTCCGTTC  AAATCCTAAG  CTTGAGAAGC  TTCGTGTACT  TTTCTGTTTC
100%
Conservation
0%
```

```
                                       1,840                          1,860                          1,880
tm-2 susc    acaaaagacc  cttccatatt  ttctcatatg  gcttgttttg  acttcaaatt  gttgcacaca  ttggttgtag  1887
Tm-2         gcaaaagacc  cttccatatt  ttctcatatg  gcttattttg  acttcaaatt  gttgcacaca  ttggttgtag  1886
Tm-2^2       gcaaaagacc  cttccatatt  ttctcatatg  gcttattttg  acttcaaatt  gttgcacaca  ttggttgtag  1887
Merlice-RC   GCAAAAGACC  CTTCCATATT  TTCTCATATG  GCTTATTTTG  ACTTCAAATT  GTTGCACACA  TTGGTTGTAG  1888
Consensus    GCAAAAGACC  CTTCCATATT  TTCTCATATG  GCTTATTTTG  ACTTCAAATT  GTTGCACACA  TTGGTTGTAG
100%
Conservation
0%
```

```
                          1,900                          1,920                          1,940                          1,960
tm-2 susc    tcatgtctca  aagttttcaa  gcatatgtca  ctatcccaag  caaatttggg  aacatgactt  gcttacgcta  1957
Tm-2         tcatgtctca  aagttttcaa  gcatatgtca  ctatcccaag  caaatttggg  aacatgactt  gcttacgcta  1956
Tm-2^2       tcatgtctca  aagttttcaa  gcatatgtca  ctatcccaag  caaatttggg  aacatgactt  gcttacgcta  1957
Merlice-RC   TCATGTCTCA  AAGTTTTCAA  GCATATGTCA  CTATCCCAAG  CAAATTTGGG  AACATGACTT  GCTTACGCTA  1958
Consensus    TCATGTCTCA  AAGTTTTCAA  GCATATGTCA  CTATCCCAAG  CAAATTTGGG  AACATGACTT  GCTTACGCTA
100%
Conservation
0%
```

```
                                       1,980                          2,000                          2,020
tm-2 susc    tctgaaattg  gaggggaata  tttgtggaaa  actgccaaat  agtattgtca  agctcacacg  tctagagacc  2027
Tm-2         tctgagattg  gaggggaata  tttgtggaaa  actgccaaat  agtattgtca  agctcacacg  tctagagacc  2026
Tm-2^2       tctgagattg  gaggggaata  tttgtggaaa  actgccaaat  agtattgtca  agctcacacg  tctagagacc  2027
Merlice-RC   TCTGAGATTG  GAGGGGAATA  TTTGTGGAAA  ACTGCCAAAT  AGTATTGTCA  AGCTCACACG  TCTAGAGACC  2028
Consensus    TCTGAGATTG  GAGGGGAATA  TTTGTGGAAA  ACTGCCAAAT  AGTATTGTCA  AGCTCACACG  TCTAGAGACC
100%
Conservation
0%
```

```
                          2,040                          2,060                          2,080                          2,100
tm-2 susc    atagacattg  atcgacgtag  cctcattcaa  cttccttctg  gtgtttggga  gtctaaacat  ttgagacatc  2097
Tm-2         atagacattg  atcgacgtag  cctcattcaa  cctccttctg  gtgtttggga  gtctaaacat  ttgagacatc  2096
Tm-2^2       atagacattg  atcgacgtag  cctcattcaa  cctccttctg  gtgtttggga  gtctaaacat  ttgagacatc  2097
Merlice-RC   ATAGACATTG  ATCGACGTAG  CCTCATTCAA  CCTCCTTCTG  GTGTTTGGGA  GTCTAAACAT  TTGAGACATC  2098
Consensus    ATAGACATTG  ATCGACGTAG  CCTCATTCAA  CCTCCTTCTG  GTGTTTGGGA  GTCTAAACAT  TTGAGACATC
100%
Conservation
0%
```

```
                        2,120                  2,140                  2,160
tm-2 susc   tttgttatag agattatgga caagcatgta acagttgctt ttctataagc tcattttacc caaacattta 2167
Tm-2        tttgttatag agattatgga caagcatgta acagttgctt ttctataagc tcattttacc caaatattta 2166
Tm-2^2      tttgttatag agattatgga caagcatgta acagttgctt ttctataagc tcattttacc caaatattta 2167
Merlice-RC  TTTGTTATAG AGATTATGGA CAAGCATGTA ACAGTTGCTT TTCTATAAGC TCATTTTACC CAAATATTTA 2168
Consensus   TTTGTTATAG AGATTATGGA CAAGCATGTA ACAGTTGCTT TTCTATAAGC TCATTTTACC CAAATATTTA
            100%
Conservation
            0%
            2,180                  2,200                  2,220                  2,240
tm-2 susc   ctcattgcat cctaacaatc tacaaacctt gatgtggata cctgataaat tttttgaacc gaggttgttg 2237
Tm-2        ctcattgcat cctaacaatc tacaaacctt gatgtggata cctgataaat tttttgaacc gaggttgttg 2236
Tm-2^2      ctcattgcat cctaacaatc tacaaacctt gatgtggata cctgataaat tttttgaacc gaggttgttg 2237
Merlice-RC  CTCATTGCAT CCTAACAATC TACAAACCTT GATGTGGATA CCTGATAAAT TTTTTGAACC GAGGTTGTTG 2238
Consensus   CTCATTGCAT CCTAACAATC TACAAACCTT GATGTGGATA CCTGATAAAT TTTTTGAACC GAGGTTGTTG
            100%
Conservation
            0%
                        2,260                  2,280                  2,300
tm-2 susc   caccgattga tcaatttaag aaaactgggt atactgggag tgtccaattc aaccgttaag atattatcaa 2307
Tm-2        caccgattga tcaatttaag aaaactgggt atactgggag tgtccaattc taccgttaag atgttatcaa 2306
Tm-2^2      caccgattga tcaatttaag aaaactgggt atactgggag tgtccaattc taccgttaag atgttatcaa 2307
Merlice-RC  CACCGATTGA TCAATTTAAG AAAACTGGGT ATACTGGGAG TGTCCAATTC TACCGTTAAG ATGTTATCAA 2308
Consensus   CACCGATTGA TCAATTTAAG AAAACTGGGT ATACTGGGAG TGTCCAATTC TACCGTTAAG ATGTTATCAA
            100%
Conservation
            0%
            2,320                  2,340                  2,360                  2,380
tm-2 susc   catgtcgccc tgtgccaaag gcgctaaagg ttctgaagct caggttttc agtgatccga gtgagcaaat 2377
Tm-2        tatttagccc tgtgctcaag gcgctggagg ttctgaagct cagtttttcc agtgacccga gtgaacaaat 2376
Tm-2^2      tatttagccc tgtgcttaag gcgctggagg ttctgaagct cagtttttcc agtgacccga gtgaacaaat 2377
Merlice-RC  TATTTAGCCC TGTGCTTAAG GCGCTGGAGG TTCTGAAGCT CAGTTTTTCC AGTGACCCGA GTGAACAAAT 2378
Consensus   TATTTAGCCC TGTGCTTAAG GCGCTGGAGG TTCTGAAGCT CAGTTTTTCC AGTGACCCGA GTGAACAAAT
            100%
Conservation
            0%
                        2,400                  2,420                  2,440
tm-2 susc   aaacttgtca tcctatccaa aaattgttaa gttgcatttg aatgttgaca gaacaatagc cttgaactct 2447
Tm-2        aaagttgtca tcgtatccac atattgctaa gttgcatttg aatgttaaca gaacaatggc cttgaactct 2446
Tm-2^2      aaagttgtca tcgtatccac atattgctaa gttgcatttg aatgttaaca gaacaatggc cttgaactct 2447
Merlice-RC  AAAGTTGTCA TCGTATCCAC ATATTGCTAA GTTGCATTTG AATGTTAACA GAACAATGGC CTTGAACTCT 2448
Consensus   AAAGTTGTCA TCGTATCCAC ATATTGCTAA GTTGCATTTG AATGTTAACA GAACAATGGC CTTGAACTCT
            100%
Conservation
            0%
            2,460                  2,480                  2,500                  2,520
tm-2 susc   gaagcattcc ctccaaatat tatcaagctt actcttgtct gctttatggt agacagttgt ctactggcag 2517
Tm-2        caatcatttc ctccaaatct catcaagctt actctagcca actttacggt agaccgttat atactggcag 2516
Tm-2^2      caatcatttc ctccaaatct catcaagctt actctagcct actttagtgt agaccgttat atactggcag 2517
Merlice-RC  CAATCATTTC CTCCAAATCT CATCAAGCTT ACTCTTGTCT GCTTTAGTGT AGACCGTTAT ATACTGGCAG 2518
Consensus   CAATCATTTC CTCCAAATCT CATCAAGCTT ACTCTNGNCT NCTTTAGNGT AGACCGTTAT ATACTGGCAG
            100%
Conservation
            0%
                        2,540                  2,560                  2,580
tm-2 susc   tgcttaagac attacccaaa ttaagaaaac ttaaaatggt catctgcaag tataatgaag aaaagatggc 2587
Tm-2        tacttaagac atttcccaaa ttaagaaaac ttaaaatgtt catctgcaag tataatgaag aaaagatgga 2586
Tm-2^2      tacttaagac atttcccaaa ttaagaaaac ttaaaatgtt catctgcaag tataatgaag aaaagatgga 2587
Merlice-RC  TACTTAAGAC ATTTCCCAAA TTAAGAAAAC TTAAAATGTT CATCTGCAAG TATAATGAAG AAAAGATGGA 2588
Consensus   TACTTAAGAC ATTTCCCAAA TTAAGAAAAC TTAAAATGTT CATCTGCAAG TATAATGAAG AAAAGATGGA
            100%
Conservation
            0%
            2,600                  2,620                  2,640                  2,660
tm-2 susc   tctctcgggc gaggcaaatg gttatagctt tccgcaactt gaagttttgc atattcatag cccgaatggg 2657
Tm-2        tctctcgggc gaggcaaatg gttatagctt tccgcaactt gaagttttgc atattcatag cccgaatggg 2656
Tm-2^2      tctctcgggc gaggcaaatg gttatagctt tccgcaactt gaagttttgc atattcatag cccgaatggg 2657
Merlice-RC  TCTCTCGGGC GAGGCAAATG GTTATAGCTT TCCGCAACTT GAAGTTTTGC ATATTCATAG CCCGAATGGG 2658
Consensus   TCTCTCGGGC GAGGCAAATG GTTATAGCTT TCCGCAACTT GAAGTTTTGC ATATTCATAG CCCGAATGGG
            100%
Conservation
            0%
                        2,680                  2,700                  2,720
tm-2 susc   ttgtctgaag taacatgcac ggatgatgtc agtatgccca aattgaaaaa gctgttactt acaggattcc 2727
Tm-2        ttgtctgaag taacgtgcac ggatgatgtc agtatgccca aattgaaaaa gctgttactt acaggattcc 2726
Tm-2^2      ttgtctgaag taacgtgcac ggatgatgtc agtatgccca aattgaaaaa gctgttactt acaggattcc 2727
Merlice-RC  TTGTCTGAAG TAACGTGCAC GGATGATGTC AGTATGCCCA AATTGAAAAA GCTGTTACTT ACAGGATTCC 2728
Consensus   TTGTCTGAAG TAACGTGCAC GGATGATGTC AGTATGCCCA AATTGAAAAA GCTGTTACTT ACAGGATTCC
            100%
Conservation
            0%
            2,740                  2,760                  2,780                  2,800
tm-2 susc   attgcggaat cagtttatcg gaacggctta aaaagctgag taaatgaaca tctcaacagg tcagtttgct 2797
Tm-2        attgccgaat cagtttatcg gaacggctta aaaagctgag taaatgaaca tctcaacagg tcagtttgct 2796
Tm-2^2      attgccgaat cagtttatcg gaacggctta aaaagctgag taaatgaaca tctcaacagg tcagtttgct 2797
Merlice-RC  ATTGCCGAAT CAGTTTATCG GAACGGCTTA AAAAGCTGAG TAAATGAACA TCTCAACAGG TCAGTTTGCT 2798
Consensus   ATTGCCGAAT CAGTTTATCG GAACGGCTTA AAAAGCTGAG TAAATGAACA TCTCAACAGG TCAGTTTGCT
            100%
Conservation
            0%
```

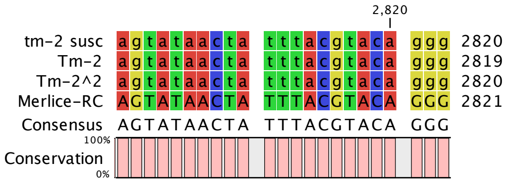
2,820
tm-2 susc agtataacta tttacgtaca ggg 2820
Tm-2 agtataacta tttacgtaca ggg 2819
Tm-2^2 agtataacta tttacgtaca ggg 2820
Merlice-RC AGTATAACTA TTTACGTACA GGG 2821
Consensus AGTATAACTA TTTACGTACA GGG
Conservation
100%
0%
