# Supplementary material for: Breeding Has Increased the Diversity of Cultivated Tomato in The Netherlands
Source: Front Plant Sci. 2019 Dec 20;10:1606. doi: 10.3389/fpls.2019.01606 (PMC6932954; doi:10.3389/fpls.2019.01606)
Supplement: Table S1 — List of studied tomato varieties. The varieties are sorted according to their years of commercial introduction in The Netherlands according to the Dutch Variety Register of the Raad voor Plantenrassen (https://nederlandsrassenregister.nl/). [file Table_1.pdf]

Table S1.

List of studied tomato varieties. The varieties are sorted according to their years of commercial introduction in The Netherlands according to the Dutch Variety Register of the Raad voor Plantenrassen (https://nederlandsrassenregister.nl/). For the 1950s we had 6 varieties, and for the subsequent decades 11, 12, 15, 15, 16, and 15 varieties.

Cultivar name	Year	Mean fruit weight, g	Fruit colour
Extase	1950	71	red
Premier	1950	83	red
Renova	1950	69	red
Robar	1950	71	red
Pygmee	1956	56	red
Nunhem´s Tuckqueen	1957	78	red
Allround	1960	54	red
No.59	1960	69	red
Superieur	1961	51	red
Roda	1962	77	red
6289 Rode	1963	66	red
Osiris	1964	66	red
Titan	1964	56	red
Rovic	1965	74	red
Moneydor	1966	59	red
Moneymar	1966	63	red
Bleke Raak	1968	69	red
V 121	1970	70	red
Roda Compacta	1971	47	red
Moneymaker	1972	62	red
Sonatine	1974	52	red
Sonato	1974	62	red
Mecano VF	1975	62	red
Ventura FR	1975	37	red
Diego	1978	90	red
Coronation	1979	58	red
Dombito	1979	133	red
Shirley	1979	44	red
Splendid	1979	60	red
Abunda	1981	83	red
Amfora	1981	92	red
Flaneur	1981	61	red
Robin svfs	1981	156	red
Novita	1982	66	red
Sierra	1982	116	red
Bornia	1983	104	red
Darus	1983	145	red

Camona	1985	82	red
Matador	1987	97	red
Ramy	1987	146	red
Start	1987	132	red
Isola	1988	32	red
Multiset	1988	66	red
Cherry Wonder	1989	16	red
Majorca	1991	100	red
Recento	1991	136	red
Tomosa	1991	84	red
Enchantment	1992	79	red
Optima	1992	311	red
Sirocco	1992	53	red
Trajan	1992	-	red
Dario	1993	88	red
Favorita	1993	13	red
Jamaica	1993	139	red
Apollo	1994	180	red
Cherokee	1995	16	red
Aromata	1996	87	red
Belle	1999	159	red
Starfighter	1999	120	red
Sunstream	2001	14	red
Bolzano	2002	79	orange
Olmeca	2004	84	brown
Amoroso (72-116 RZ)	2005	29	red
Arawak	2005	252	red
Lorenzo	2005	88	yellow
Mini Star	2005	9	red
Elanto (72-372 RZ)	2007	125	red
Juanita	2007	9	red
Philovita	2007	13	red
Roterno (72-230 RZ)	2007	67	red
Santorange	2007	9	orange
Tomimaru Muchoo	2007	170	red
Arvento (72-375 RZ)	2008	105	red
Starbuck	2008	234	red
Angelle	2009	12	red
Tourance	2010	92	red
DRK 936	2011	61	red
Komeett	2011	-	red
Sassari (72-138 RZ)	2011	14	red
Sweetelle	2011	9	red
Bambelo,oranje	2013	13	orange
Diamantino	2013	111	red
Merlice	2013	131	red

Climstar	2014	159	red
Prolyco 2	2014	31	red
Romanella	2014	123	red
Wasino (72-155 RZ)	2014	33	red
DR7024TS	2015	259	red
Vacetto (72-164 RZ)	2015	127	red
SV0948TS	2016	13	yellow
