# Supplementary material for: Breeding Has Increased the Diversity of Cultivated Tomato in The Netherlands
Source: Front Plant Sci. 2019 Dec 20;10:1606. doi: 10.3389/fpls.2019.01606 (PMC6932954; doi:10.3389/fpls.2019.01606)
Supplement: Table S2 — Trends for basic flavor parameters. Mann-Kendall trend statistics for all the varieties studied (n = 90), and for the non-cherry (medium-sized and large fruits, >30 g/fruit) varieties. Trend coefficient S estimates strength and directionality of a trend. The statistical significance is represented by a P-value. Trends are estimated for the entire collection of 90 varieties, and for a sub-set of medium-sized and large fruited varieties (Non-cherry). SSC – soluble solid content, equivalent of sugar content in tomato, TA – titratable acidity, Firmness – firmness of fruit pericarp, Juiciness – amount of liquid released from tomato fruit pericarp. [file Table_2.pdf]

Table S2. Trends for basic flavor parameters. Mann-Kendall trend statistics for all the varieties studied (n = 90), and for the non-cherry (medium-sized and large fruits, >30 g/fruit) varieties. Trend coefficient S estimates strength and directionality of a trend. The statistical significance is represented by a P-value. Trends are estimated for the entire collection of 90 varieties, and for a sub-set of medium-sized and large fruited varieties (Non-cherry). SSC – soluble solid content, equivalent of sugar content in tomato, TA – titratable acidity, Firmness – firmness of fruit pericarp, Juiciness – amount of liquid released from tomato fruit pericarp.

**All varieties**	**SSC**	**TA**	**SSC/TA**	**Firmness**	**Juiciness**
S	781	-299	1633	874	-499
**trend**	Increase		Increase	Increase	
**P-value**	0.005	0.274	2.18E-09	0.002	0.072

**Non-Cherry**	**SSC**	**TA**	**SSC/TA**	**Firmness**	**Juiciness**
S	196	-772	1111	251	-737
**trend**		Decrease	Increase		Decrease
**P-value**	0.38	0.0003	2.21E-07	0.25	0.0007
