# Supplementary material for: Breeding Has Increased the Diversity of Cultivated Tomato in The Netherlands
Source: Front Plant Sci. 2019 Dec 20;10:1606. doi: 10.3389/fpls.2019.01606 (PMC6932954; doi:10.3389/fpls.2019.01606)
Supplement: Table S3 — Volatile compounds which had a statistically significant quantitative trends in fruits of tomato varieties introduced from 1950 till 2016. Directionality and strength of trends is represented by Mann-Kendall trend coefficient S with positive and negative values indicating an increase and decrease, respectivelly, in concentration during the period of time studied. Trends are estimated in the entire collection of 90 varieties and in a sub-set of medium and large fruited varieties (Non-cherry). P-values represent significance of the trend [file Table_3.pdf]

Table S3. Volatile compounds which had a statistically significant quantitative trends in fruits of tomato varieties introduced from 1950 till 2016. Directionality and strength of trends is represented by Mann-Kendall trend coefficient S with positive and negative values indicating an increase and decrease, respectively, in concentration during the period of time studied. Trends are estimated in the entire collection of 90 varieties and in a sub-set of medium and large fruited varieties (Non-cherry). P-values represent significance of the trend estimates.

**Volatile compound**	**Biosynthetic group**	**Mann-Kendall trend test**				**Aroma descriptors in tomato fruit**
		**S (All)**	**p-value**	**S (Non-cherry)**	**p-value**	
2-Phenylethanol	Phenolic	1986	2E-12	1357	5.5E-10	floral (*28*, *32*), alcohol, nutty(*30*), tropical & fruity (*31*)
Phenylacetaldehyde	Phenolic	1818	1.2E-10	1173	8.3E-08	floral(*26*, *32*), honey(*27*), ginger, rosy(*26*), beeswax(*42*)
2-Phenylnitroethane	Phenolic	1808	1.5E-10	1402	3.3E-10	sweet-floral, warm-spicy (*43*)
2-Octenal, (E)-	Lipid	904	0.00137	-505	0.0238	nutty, cooked, raw peanut(*28*), fatty, green, leafy, mouldy(*26*), citrus (*42*
2,4-Decadienal, (E,E)-	Lipid	648	0.02186	-732	0.00104	earthy, musty(*29*), fatty (*42*)
1-Pentanol	Lipid	700	0.01176			
alpha-Terpineol	Terpenoid	-708	0.01223	-443	0.04744	
3-Methylbutanal	BCAA	-740	0.00882	-756	0.00055	stale, rotten, nutty (*26*, *30*, *31*), unpleasant(*27*), rancid, musty(*26*), malty (*42*)
2,4-Heptadienal, (E,E)-	Lipid	-787	0.00534	-732	0.00104	boiled potato(*28*)
Methylthioacetaldehyde	Sulphurous AA	-787	0.0046	-780	0.00048	
p-Mentha-1,3-dien-7-al	Terpenoid	-802	0.00453	-572	0.01044	
3-Methyl-1-butanol	BCAA	-848	0.00269	-799	0.00026	sweet, fresh(*30*)
p-Menth-1-en-9-al	Terpenoid	-982	0.00051	-794	0.00038	
2-Methyl-1-butanol	BCAA	-1103	9.4E-05	-737	0.00076	
Methyl salicylate	Phenylpropanoid	-1186	2.7E-05	-762	0.0005	oily(*27*), vegetal, minty, rancid(*26*)
beta-Phellandrene	Terpenoid	-1188	2.6E-05	-927	3.3E-05	chemical(*27*)
Methional	Sulphurous AA	-1218	1.2E-05	-1141	1.8E-07	potato, pungent(*29*), cooked potato(*26*, *27*)
Nonane	Lipid	-1238	1.2E-05	-1141	1.8E-07	
p-Cymene	Terpenoid	-1250	9.6E-06	-732	0.00104	
Eugenol	Phenylpropanoid	-1304	3.9E-06	-731	0.00084	cloves(*42*), medical, phenolic(*44*)
Guaiacol	Phenylpropanoid	-1336	2.2E-06	-809	0.00022	smoky(*21*, *42*), pharmaceutical(*21*), off-flavour(*45*)
Salicylaldehyde	Phenylpropanoid	-1574	2.5E-08	-974	8.6E-06	green(*32*)
