# Supplementary material for: Breeding Has Increased the Diversity of Cultivated Tomato in The Netherlands
Source: Front Plant Sci. 2019 Dec 20;10:1606. doi: 10.3389/fpls.2019.01606 (PMC6932954; doi:10.3389/fpls.2019.01606)
Supplement: Supplementary file 17 [file Presentation_1.pdf]

Supplementary Materials for

Breeding has increased the diversity of cultivated tomato in The Netherlands

Henk J. Schouten, Yury Tikunov, Wouter Verkerke, Richard Finkers, Arnaud Bovy, Yuling Bai, Richard G.F. Visser

Correspondence to: henk.schouten@wur.nl

Supplementary Text

Validation of the trend in fruit size diversification

As described in the main text, we observed diversification of fruit sizes since the 1990s in the 90 tomato varieties. In order to validate this trend, we evaluated additional historical fruit size data of 284 varieties (including the 90 experimental varieties) . Fruit size class data (classes from 1 to 9) registered from 1950 till 2016 (~40 per decade) were derived from the official Dutch variety registry. The fruit size classes were converted to fruit weight (g) using a regression of the experimental fruit weight data to their corresponding fruit sizes classes as recorded in the variety registry (Fig. S4B). As the result, among the 284 varieties, the proportion of varieties with an average weight 73±22 g per fruit has been gradually declining in course of time, whereas small fruited varieties with 15 ±8 g fruits, and varieties with large fruits (> 90 g) have been registered more frequently in the last three decades (Fig. S4C). This corresponds with our observation in the experiment with the 90 varieties.

Identification of the most variable volatile compounds

To identify the volatile compounds that showed the highest quantitative variation, 69 annotated volatiles (Data S4) were subjected to an unsupervised multivariate data analysis – Principal Components Analysis (PCA) (Fig. S6). Principal component 1 (PC1) explains 41% of the variation between the tomato varieties. This variation is due to different average concentrations of phenolic and phenylpropanoid volatiles: a group of varieties encircled in red have higher fruit concentrations of phenolic volatiles (e.g. 2-phenylethanol, phenylacetaldehyde, 2-phenylnitroethane: sweet, floral, fruity aromas (Table S2) and lower concentrations of phenylpropanoid volatiles (guaiacol, methyl salicylate and eugenol: smoky, phenolic, medical aromas) compared to the varieties encircled in green. In addition, PC2 which accounts for 13% of the variation, shows varieties with high concentration of another type of volatiles – derived from catabolism of branched-chain and sulphur-containing amino acids (encircled in blue). Interestingly, a larger proportion of the early decades' varieties are located in the area of high concentrations of phenylpropanoid and branched-chain/sulphurous amino acid volatile derivatives.
